# Lignin as a Sustainable Building Block for Electrochemical Energy Devices: A Critical Review

**DOI:** 10.3390/polym18212669

**Published:** 2026-10-28

**Authors:** Pablo Reyes-Contreras, María Graciela Aguayo, Rosario del P. Castillo, Regis Teixeira Mendonça

**Affiliations:** 1 Centro de Biotecnología, Universidad de Concepción, Concepción 4030000, Chile; rosariocastillo@udec.cl (R.d.P.C.); rteixeira@udec.cl (R.T.M.); 2 Centro de Innovación en Tecnología y Diseño de Materiales para el Entorno Construido—CIMAc, Avenida Libertador Bernardo O’Higgins 3363, Santiago 9170022, Chile; 3 Departamento de Ingeniería de Procesos y Bioproductos, Facultad de Ingeniería, Universidad del Bío-Bío, Avenida Collao 1202, Concepción 4051381, Chile; 4 Centro Nacional de Excelencia Para la Industria de la Madera (CENAMAD), Pontificia Universidad Católica de Chile, Vicuña Mackenna 4860, Santiago 7820436, Chile; 5 Departamento de Análisis Instrumental, Facultad de Farmacia, Universidad de Concepción, Concepción 4030000, Chile; 6 Facultad de Ciencias Forestales, Universidad de Concepción, Concepción 4030000, Chile

**Keywords:** lignin, modification, electrochemical energy, energy storage

## Abstract

Lignin, the most abundant aromatic biopolymer on Earth, has gained significant attention as a sustainable precursor for materials used in advanced energy storage technologies. Its intrinsic chemical complexity, featuring phenolic, methoxy, and quinone-like functionalities, enables multiple valorization pathways, including the production of carbon materials, redox-active polymers, polymer electrolytes, and functional binders. Recent advances in lignin extraction, fractionation, targeted chemical modification, and controlled carbonization have significantly broadened the functional scope and electrochemical performance of lignin-based components across a wide range of systems, including batteries, supercapacitors, and hybrid devices. This review consolidates recent progress in the development of lignin-derived materials for electrochemical energy storage, emphasizing structure–function relationships and the influence of lignin source and processing route on final material properties. Finally, we outline emerging prospects for integrating lignin-based materials into next-generation sustainable energy storage systems and present a roadmap for accelerating future scientific and industrial development.

## 1. Introduction

Lignocellulosic biomass derived from forest resources is among the most abundant and renewable carbon sources on Earth, providing a strategic feedstock for the development of sustainable materials and energy technologies. Within this complex biopolymeric matrix, lignin is the principal natural source of aromatic structures and typically accounts for approximately 15–30% of plant biomass, depending on the botanical source and biomass type [1,2,3]. Its high carbon content, aromatic nature, and abundance make lignin particularly attractive as a renewable precursor for developing functional carbon materials, including electrode materials for energy storage applications [3,4]. Despite its chemical richness, characterized by phenolic, methoxy, and other oxygen-containing functionalities, lignin remains substantially underutilized in high-value applications. A large fraction of industrial lignin is still primarily used for process heat and energy generation, particularly in the pulp and paper industry, rather than being converted into higher-value products [5].

The increasing deployment of renewable energy technologies, particularly solar and wind power, has intensified demand for efficient, reliable, and scalable energy storage systems to mitigate the intermittency of these resources [6]. Electrochemical energy storage (EES) technologies, including lithium-ion batteries and supercapacitors, have become important components of modern energy systems because of their complementary energy and power characteristics, high efficiency, and broad applicability, ranging from portable electronics to stationary and grid-scale storage [7,8,9]. In this context, lignin-derived materials have attracted increasing attention as potentially sustainable alternatives to conventional carbon-based electrode materials. Lignin’s aromatic, carbon-rich structure provides a favorable molecular framework for conversion into porous, functional carbon structures, while its chemical functionalities enable tailoring of surface chemistry, morphology, and interfacial properties.

Fractionation, chemical functionalization, and controlled thermal conversion can further tailor the performance of lignin-based materials. These strategies can modify molecular-weight distribution, functional group composition, crosslinking behavior, pore structure, and surface chemistry, thereby influencing the physicochemical and electrochemical properties of the resulting materials [10,11,12]. Depending on the processing strategy, lignin can be employed as a precursor for carbonaceous electrodes, functional components, binders, or building blocks for hybrid and composite electrode architectures [10,11]. However, the intrinsic structural heterogeneity and compositional variability of lignin, arising from both the biomass source and the extraction process, remain major challenges to achieving reproducible material properties. In addition, the limited intrinsic electrical conductivity of unmodified lignin restricts its direct application in electrochemical devices, requiring its conversion into conductive carbon structures or integration with electrically conductive phases [4,13,14]. Consequently, controlled fractionation and environmentally responsible chemical and thermal modification strategies are essential for establishing reproducible structure–property relationships and enabling the scalable production of lignin-derived electrode materials.

Although substantial progress has been made in the development of lignin-derived materials for EES applications, the relationships among lignin feedstock characteristics, extraction and processing history, chemical structure, conversion pathways, and electrochemical performance remain insufficiently integrated in the literature [3,10,12]. Many studies have focused on specific lignin sources, processing routes, or individual EES technologies, making it difficult to establish general structure–processing–property relationships that are applicable across different lignin feedstocks and material platforms. Addressing this gap is particularly important because variations in lignin origin and processing can substantially affect the molecular structure, functional group distribution, thermal behavior, carbonization characteristics, porosity, and ultimately the electrochemical performance.

This review critically examines lignin as a sustainable building block and precursor for electrochemical energy storage materials, with particular emphasis on the relationships between lignin structure, extraction and fractionation strategies, processing pathways, and material performance. Lignins obtained from different biomass sources and extraction processes are first discussed in terms of their structural and physicochemical characteristics. Their subsequent transformation into functional carbon materials and hybrid architectures for various EES technologies is evaluated, with an emphasis on the mechanisms that link processing conditions and material properties to electrochemical performance. Finally, the major limitations, emerging strategies, and future research directions required to translate lignin-based materials from laboratory-scale demonstrations into reproducible, scalable, and high-performance energy storage technologies are identified.

## 2. Lignin: Structure, Chemistry and Biosynthesis

Lignin constitutes 15–30% of biomass and plays a vital role in reinforcing the plant cell wall and imparting rigidity to plant tissues [15,16]. Lignin is a heterogeneous aromatic polymer composed of phenylpropanoid units derived from the oxidative polymerization of monolignols, such as *p*-coumaryl, coniferyl, and sinapyl alcohols. These precursors give rise to *p*-hydroxyphenyl (H), guaiacyl (G), and syringyl (S) units, respectively. The primary distinction among these units lies in the number of methoxy groups attached to the aromatic ring: H units contain no methoxy substituents, G units contain one methoxy group, and S units contain two methoxy groups. These units are interconnected through carbon–carbon (5–5, β–5, β–1, and β–β) and ether (β–O–4, α–O–4, and 4–O–5) linkages [16,17,18]. The abundance and distribution of lignin intermonomeric linkages vary among plant taxa, cell types, and cell wall layers. Softwoods predominantly contain G-type lignin, hardwoods are characterized by SG-type lignin, and grasses exhibit HGS-type lignin [17,19,20]. Among these linkages, the β–O–4 bond is the most prevalent in native lignin, typically accounting for approximately 50% of the linkages formed during biosynthesis. Moreover, it is one of the most labile bonds and is readily cleaved by most pretreatment and depolymerization processes [21,22,23].

In addition to its structural function, the chemical architecture of lignin underpins its redox activity, which is particularly relevant to electrochemical energy storage (EES). The phenolic hydroxyl groups associated with the H, G, and S units serve as crucial redox-active functionalities in lignin, with catechol-type structures being notably susceptible to reversible oxidation to quinone/semiquinone species via coupled proton and electron transfer processes [12,24]. This inherent phenol–quinone redox chemistry allows lignin to engage in charge-storage processes directly and has been utilized in lignin-based organic electrodes and supercapacitors [11,12,24]. The density and accessibility of these redox-active phenolic groups depend on lignin’s structure, botanical origin, and processing history, which can significantly affect its electrochemical response. For instance, enhancing the phenolic/catechol content of technical lignin through chemical functionalization has been demonstrated to markedly increase its charge storage capacity [24]. Conversely, methoxy groups, which differentiate G- and S-type units, should not be considered primary redox centers; rather, their substitution patterns can alter the electronic environment and oxidation behavior of the aromatic units, thereby affecting the overall redox characteristics of lignin [11,12,24]. Consequently, the distribution of phenolic and quinone-related functionalities, along with the substitution pattern of the aromatic units, establishes a direct structure–redox relationship that provides a fundamental rationale for employing lignin as a bio-derived redox-active material in EES systems [24].

## 3. Technical Lignins from Industrial Fractionation Processes

Lignin is not a single chemical substance; its structure and physicochemical properties, including molecular weight, sulfur content, purity, functional-group density, and thermal behavior, depend strongly on its botanical origin and isolation method. Lignin can be obtained as native or near native lignin from plant cell walls, including milled wood lignin (MWL), cellulolytic enzyme lignin (CEL), and enzymatic mild-acidolysis lignin (EMAL), or as analytical lignin fractions, such as Klason lignin [25,26,27]. However, these materials are primarily used for structural characterization and are generally not available at the scale required for industrial application. In contrast, lignins recovered from pulping and lignocellulosic biorefinery streams are commonly called technical lignins. The four major industrial technical lignins are Kraft lignin, lignosulfonates produced by sulfite pulping, organosolv lignin, and soda lignin. Each is generated through a distinct fractionation route and consequently displays a unique physicochemical profile that affects its electrochemical performance [28,29].

### 3.1. Kraft Lignin

Kraft lignin is produced as a byproduct of the predominant alkaline pulping process, which accounts for approximately 85% of worldwide pulp production. In this process, biomass is treated with a mixture of sodium hydroxide and sodium sulfide under high-temperature and high-pressure conditions. The lignin is subsequently precipitated from the black liquor by acidification, using methods such as LignoBoost, LignoForce, or Sequential Liquid-Lignin Recovery and Purification (SLRP). This procedure significantly cleaves β–O–4 bonds, introduces residual thiol/sulfide groups, and enhances aromatic condensation, resulting in lignin with increased molecular weight and polydispersity, and a sulfur content ranging from 1% to 3% [30,31]. Due to its low cost, large-scale availability in thousands of metric tons, and high carbon content, Kraft lignin is favored as a raw material for producing activated carbons and carbon nanofibers, which are utilized as supercapacitor electrodes. Recent research has demonstrated that quaternized Kraft lignin can be directly employed as an electrolyte additive in aqueous zinc-ion batteries, effectively suppressing hydrogen evolution and zinc dendrite formation. Additionally, its intrinsic electrical conductivity, which is highly influenced by morphology and post-treatment, makes it a viable active material for low-power electronic devices [29,32,33].

### 3.2. Organosolv Lignin

The organosolv process employs organic solvents, such as ethanol, methanol, and acetic acid, typically in conjunction with an acid catalyst, to decompose biomass without using sulfur. This method yields high-purity, sulfur-free lignin characterized by reduced molecular weight, lower polydispersity, and significantly higher retention of β–O–4 bonds than Kraft lignin, making it structurally more similar to native lignin [34,35]. Enhanced structural uniformity and solubility in organic solvents facilitate the conversion of cellulose into high-value-added products, including membranes, nanoparticles, and electrospun fibers. Several reviews have underscored that, in contrast to sulfur-based processes such as Kraft and sulfite, the organosolv method provides higher purity and partially preserved structure. This results in activated carbons with increased surface area and microporosity after KOH activation, thereby improving hydrogen storage and capacitive performance. However, the primary limitation to energy scalability remains the elevated cost of solvent recovery [28,34,35].

### 3.3. Lignosulfonates

Lignosulfonates are produced as byproducts of the sulfite process, in which lignin undergoes direct sulfonation during cooking. This process incorporates sulfonate groups (–SO_3_^−^), which impart high solubility and hydrophilicity in water, as well as notable polyelectrolytic and surfactant properties [29,36]. In contrast to Kraft and organosolv lignin, lignosulfonates have a significantly higher sulfur content due to their covalent sulfonate groups and generally exhibit a higher ash content. Their aqueous solubility and anionic nature render them particularly advantageous as water-processable binders, dopants/templates for the in situ polymerization of polyaniline or polypyrrole, and components of electrolytic hydrogels for flexible supercapacitor applications. These materials have achieved specific capacitances of up to 553.7 F/g in lignosulfonate/polyaniline composites and 624.36 mF/cm^2^ in sodium lignosulfonate hydrogels for self-healing supercapacitors. A comparative study of lignosulfonate, partially desulfonated lignosulfonate, and desulfonated Kraft lignin revealed that the degree of sulfonation influences the aqueous processability, stability, and faradaic contribution of the composite electrode. Partial desulfonation was found to offer the optimal balance for large-scale organic battery electrodes [36,37,38].

### 3.4. Soda Lignin

Soda lignin is produced through a sulfur-free alkaline pulping process that employs sodium hydroxide (NaOH) and, occasionally, anthraquinone as a redox catalyst. This method primarily utilizes non-wood biomass, including bagasse, wheat straw, coconut husks, and agricultural waste. The absence of sulfur in this process yields lignin of higher purity than that obtained via the Kraft process, although it generally has a higher molecular weight than organosolv lignin. For instance, lignin-derived from coconut shells has been reported to have a molecular weight (Mw) of 959 g/mol for soda lignin, whereas Kraft and organosolv lignins have molecular weights of 769 g/mol and 606 g/mol, respectively, under similar conditions. Moreover, soda lignin is noted for its enhanced thermal stability and elevated glass transition temperature compared with Kraft lignin, making it advantageous for controlled carbonization in producing carbon precursors for electrodes. Its sulfur-free nature, akin to that of organosolv lignin, makes it a favorable “green” alternative to Kraft lignin, particularly when sulfur contamination in battery or supercapacitor carbon materials is a concern [39,40].

Table 1 provides a comparative summary of the main types of technical lignin, organized by production processes and the resulting physicochemical and structural characteristics. The differences among kraft, organosolv, lignosulfonate, and soda lignins arise primarily from the severity and chemical environment of the delignification process, which strongly influences the sulfur content, molecular weight, solubility, ash and carbohydrate content, and preservation of native lignin linkages.

Taken together, differences among technical lignins are not merely descriptive; they directly influence their suitability as precursors for electrochemical materials. Kraft lignin generally combines high aromaticity with substantial condensation and residual inorganic or sulfur-containing species, whereas Organosolv lignins tend to retain a less condensed structure and lower ash content. Lignosulfonates provide high water dispersibility and distinct sulfur-containing functionality, while soda lignins generally have low sulfur content but remain strongly dependent on biomass and recovery conditions. These differences can modify thermal degradation, carbon yield, pore development, heteroatom retention, and surface chemistry during subsequent carbonization and activation. Therefore, comparisons of lignin-derived electrochemical materials should not treat “lignin” as a uniform precursor. Differences in precursor origin and isolation history may contribute to variations in electrochemical performance that are otherwise attributed solely to the carbonization or activation strategy.

## 4. Fractionation and Chemical Modification Strategies

The structural heterogeneity of technical lignin, including variability in molecular weight, distribution of G/S/H units, phenolic and aliphatic hydroxyl group content, and the presence of sulfur or residual carbohydrates, constitutes the main barrier to its utilization in high-performance applications, including electrochemical energy storage [49,50]. Therefore, between lignin extraction and its conversion into functional carbonaceous materials, a conditioning step is essential to reduce heterogeneity and/or incorporate specific functional groups into the lignin-derived products. This section reviews three families of complementary strategies: (i) solvent fractionation to obtain subfractions with controlled molecular weights [51,52], (ii) nanostructuring of lignin using physically/chemically mild methods [53,54], and (iii) covalent chemical modification (carboxymethylation, sulfomethylation, esterification) aimed at introducing redox-active or hydrophilic groups [55,56,57], which is a critical aspect for its subsequent performance as an electrode material or additive in EES devices.

### 4.1. Solvent Fractionation

Sequential fractionation using organic solvents of increasing polarity, such as methanol, ethanol, acetone, ethyl acetate, and dioxane/water, facilitates the division of technical lignin into subfractions characterized by a significantly narrower Mw distribution than the original material. This process effectively reduces the polydispersity index (PDI) and concentrates specific functional groups in each fraction. Jiang et al. [49] demonstrated that sequential precipitation of hardwood Kraft lignin using solvents of decreasing polarity produces fractions with Mw ranging from approximately 1000 to over 10,000 g/mol, alongside a progressive enrichment of phenolic hydroxyl groups in the lower-molecular-weight fraction. Kim et al. [51] observed a comparable pattern in their study on the fractionation of soda lignin using ethyl acetate, methanol, acetone, and dioxane/water mixtures. They obtained fractions with Mw ranging from 1120 to 7200 Da. Through 2D HSQC-NMR analysis, it was confirmed that the relative abundance of the β–O–4 bond increased with the fraction’s molecular weight, thereby influencing its reactivity. Izaguirre et al. [52] conducted a comparative study on the fractionation of Kraft and Organosolv lignins derived from Eucalyptus globulus. They employed a sequence of eight solvents with increasing polarities: methanol, ethanol, acetone, ethyl acetate, 1-propanol, 2-propanol, butanone, and 1-butanol. The effectiveness of this method, irrespective of the initial extraction process, was confirmed using FTIR, ^31^P NMR, Py-GC/MS, TGA, and DSC analyses. The process yields low-molecular-weight fractions rich in reactive sites, making them suitable as active materials or comonomers, and high-molecular-weight fractions with enhanced thermal resistance, suitable for structural applications [52]. Similarly, Song et al. achieved comparable outcomes by fractionating lignin extracted from hybrid poplar with p-toluenesulfonic acid, thereby reducing the polydispersity index from 3.31 to 1.44 and enhancing the thermal stability of the purified fractions [58]. A recent review summarized these advancements for lignin derived from black liquor, emphasizing ethyl acetate, ethanol, and acetone as the most frequently utilized fractionation solvents at the laboratory scale, owing to their optimal balance of selectivity, cost, and environmental considerations [59].

This fractionation step is particularly relevant to the proposed experimental work, in which Kraft, Organosolv, and lignosulfonate lignin are fractionated using ethyl acetate to obtain subfractions with a Mw of between 1500 and 3000 g/mol. This range facilitates both the controlled formation of nanoparticles and access to functional groups for subsequent chemical modification.

### 4.2. Alkaline Treatment

Alkaline treatment offers a versatile route to modify lignin structure and promote partial depolymerization, with the extent and nature of the transformations strongly dependent on reaction severity and the presence or absence of an oxidizing agent. Under strongly alkaline conditions, NaOH promotes the deprotonation of phenolic and aliphatic hydroxyl groups, increasing lignin solubility and facilitating the cleavage of lignin–carbohydrate bonds and susceptible interunit linkages. In particular, the β–O–4 aryl ether linkage is one of the most reactive sites under alkaline conditions. Mechanistic studies indicate that base-catalyzed cleavage can proceed via deprotonation at the Cα position and subsequent cleavage of the C–O bond, yielding epoxide- or quinone-methide-type intermediates, depending on the lignin structure [60,61]. Experimental studies of sequential alkaline pretreatment have similarly shown that β–O–4 linkages are substantially more susceptible to degradation than β–β and β–5 C–C linkages, the latter becoming comparatively recalcitrant under repeated alkaline treatment [62].

When an oxidizing agent such as H_2_O_2_ is introduced, the alkaline environment facilitates oxidative modification in addition to base-catalyzed cleavage, promoting oxidation of benzylic and side-chain functionalities and increasing the abundance of carbonyl and carboxyl groups while further reducing lignin molecular weight. Under appropriate conditions, the oxidative cleavage of β–O–4 and Cα–Cβ bonds can generate lower-molecular-weight aromatic fragments and carboxyl-rich oligomers [63,64].

More extensive oxidation can also affect the aromatic ring itself, leading to ring-opening products such as muconic acid-type structures, as demonstrated for different Kraft and Organosolv lignins treated with H_2_O_2_-based oxidative systems [65]. Hence, alkaline and alkaline oxidative treatments should be regarded as distinct strategies: NaOH mainly promotes solubilization, deprotonation, and ether-bond cleavage, whereas H_2_O_2_ additionally enables oxidative functionalization, side-chain cleavage, and, in some cases, aromatic-ring opening. This tunability enables lignin fractions with different molecular weights and enriched phenolic, carbonyl, and carboxyl functionalities that can be further functionalized into value-added materials.

**Figure 1:**
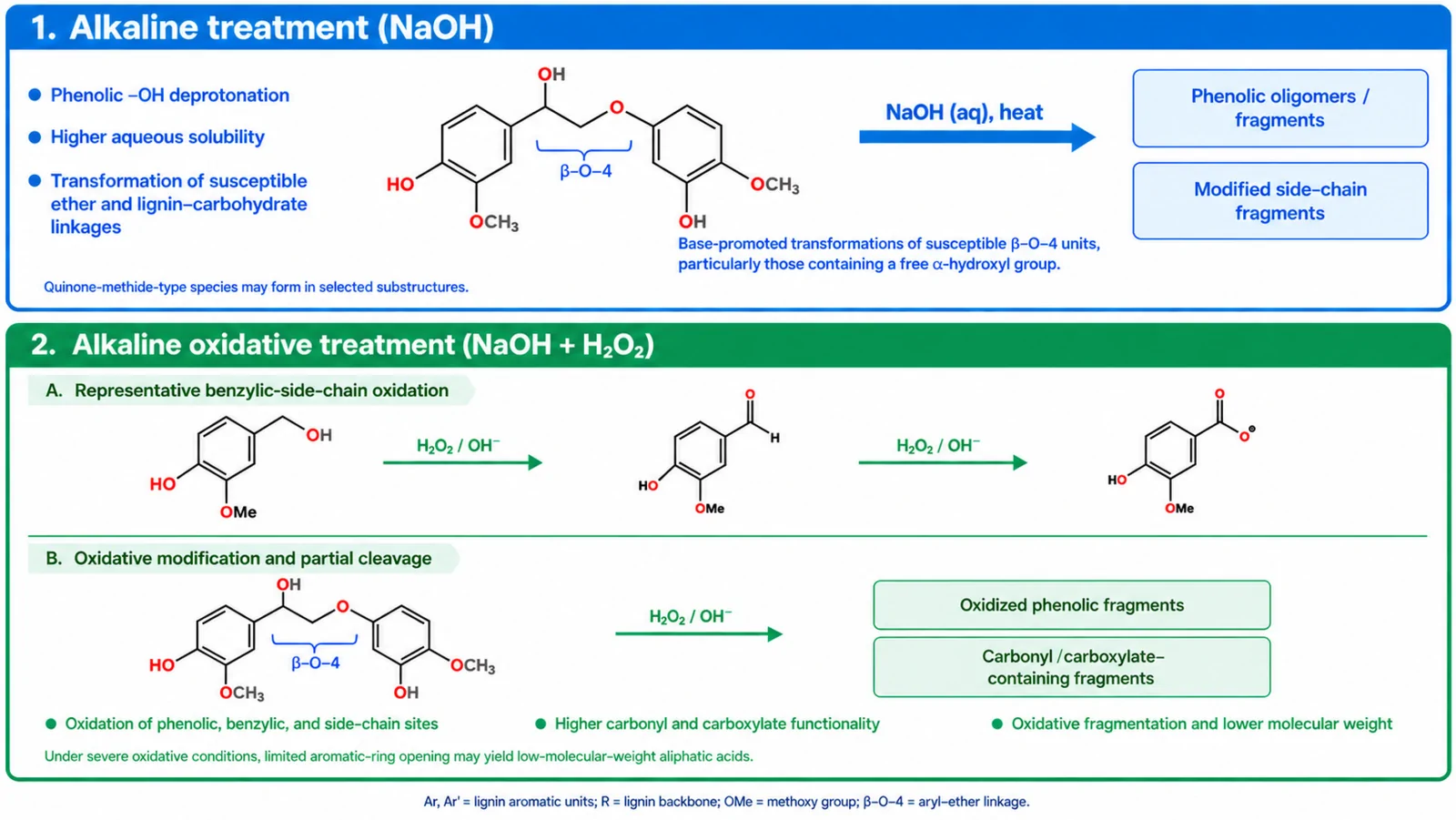
Chemical pathways of alkaline and alkaline oxidative lignin treatment.

The main chemical transformations associated with alkaline and alkaline oxidative lignin treatments are summarized in Figure 1. Under alkaline conditions, NaOH promotes deprotonation and cleavage of susceptible ether linkages, particularly β–O–4 structures, whereas the addition of H_2_O_2_ introduces oxidative pathways involving benzylic and side-chain functionalities, oxidative bond cleavage, and, under more severe conditions, aromatic ring opening.

From an application perspective, the main distinction between alkaline and alkaline-oxidative treatment is not simply the extent of lignin depolymerization but the type and distribution of functionalities generated. NaOH treatment primarily increases solubilization and exposes or generates phenolic sites, whereas adding H_2_O_2_ introduces additional carbonyl and carboxyl functionalities and can promote more extensive side-chain or aromatic-ring oxidation. These changes can improve polarity, dispersibility, and subsequent chemical reactivity, but excessive oxidation may also decrease aromatic integrity and reduce the carbon yield available for subsequent conversion into conductive carbon materials. Thus, the optimal oxidation severity depends on the intended EES function: conditions that maximize functional-group density for redox or interfacial applications may not be optimal for producing high-yield, electrically conductive carbon frameworks.

### 4.3. Functional Chemical Modification

Incorporating polar or redox-active functional groups through targeted chemical reactions is the most direct approach to modifying lignin reactivity for specific applications. These applications include its use as a precursor for activated carbon, electroactive additive, or dispersant. Three modification pathways have been extensively investigated.

#### 4.3.1. Carboxymethylation

Carboxymethylation is an effective etherification strategy for introducing anionic carboxymethyl groups into lignin, thereby modifying its solubility, charge density, reactivity, and interactions with other components. The reaction is commonly performed under alkaline conditions, using sodium hydroxide to activate lignin and sodium chloroacetate (SCA) or monochloroacetic acid as carboxymethylating agents. Under these conditions, NaOH deprotonates lignin hydroxyl groups, particularly phenolic hydroxyls, generating nucleophilic lignin phenolates that attack the electrophilic carbon of chloroacetate via an S_N_2-type substitution, forming lignin–O–CH_2_–COO^−^ groups [55,66,67]. Consequently, this modification increases the density of ionizable carboxylate groups and generally enhances lignin water dispersibility and electrostatic characteristics [66,68,69]. Carboxymethylation is not restricted to a single chemically equivalent site because lignin contains phenolic and aliphatic hydroxyl groups that differ in accessibility and reactivity. Therefore, the degree and distribution of substitution depend strongly on the lignin origin, extraction history, molecular structure, hydroxyl group content, and reaction conditions [55,70]. A recent systematic study by Andriani et al. [55] demonstrated substantial differences in carboxymethylation reactivity among eucalyptus and spruce Kraft lignins and birch and spruce Organosolv lignins, establishing a direct relationship between lignin structural features and carboxymethylation efficiency. The reaction conditions, particularly the NaOH concentration, SCA-to-lignin ratio, temperature, reaction time, and lignin concentration, further control the incorporation of carboxymethyl groups and the resulting molecular weight distribution and charge density [66,69]. Process intensification through ultrasound has also been demonstrated for LignoForce Kraft lignin, reducing the reaction time from approximately 4 h to 30 min while producing water-dispersible carboxymethylated lignins; however, high-intensity sonication can simultaneously promote the cleavage of aryl-ether linkages and modify the molecular weight distribution [69].

Recent studies have confirmed that carboxymethylation generates additional reactive sites that can facilitate crosslinking and network formation in lignin-based hydrogels, thereby improving their compatibility with polymeric matrices [71,72]. The increased density of carboxylate groups also provides additional coordination and electrostatic interaction sites for metal ions, thereby enabling the development of carboxymethylated lignin-based materials for metal capture and wastewater treatment [69,73]. Carboxymethylated lignin-based flocculants have demonstrated enhanced Cu^2+^ capture through electrostatic interactions and complexation, whereas carboxymethylation of residual lignin in lignocellulosic biomass can reduce nonproductive enzyme adsorption and improve enzymatic hydrolysis [73,74]. Recent studies have further extended this strategy to electrochemical applications, showing that carboxymethylated Kraft lignin can be obtained as an anionic, water-soluble material with a high zeta potential and properties favorable for electrode coating [67]. Thus, carboxymethylation provides a tunable route for converting relatively hydrophobic and structurally heterogeneous technical lignins into more hydrophilic and anionic functional materials, with the extent of substitution and the preservation of the lignin backbone as key parameters governing subsequent performance, including molecular-weight aromatic fragments and carboxyl-rich oligomers [68,69].

This tunability enables the selective generation of lignin fractions with altered molecular-weight distributions and enriched phenolic, carbonyl, and carboxyl functional groups, which can be exploited for chemical functionalization and the development of value-added materials.

The electrochemical relevance of carboxymethylation therefore depends on balancing functionalization with preservation of the lignin framework. Increasing carboxymethyl substitution can enhance hydrophilicity, charge density, and interaction with ionic species, but excessive modification may alter molecular conformation, reduce aromatic interactions, or introduce mass that does not directly contribute to charge storage. Importantly, improvements in dispersibility or ionic interaction should not be equated automatically with improved electrochemical performance. For EES applications, we must evaluate the benefit of carboxymethylation against resulting changes in charge-transfer resistance, ion accessibility, redox-site density, and structural stability under cycling.

#### 4.3.2. Sulfomethylation

**Figure 2:**
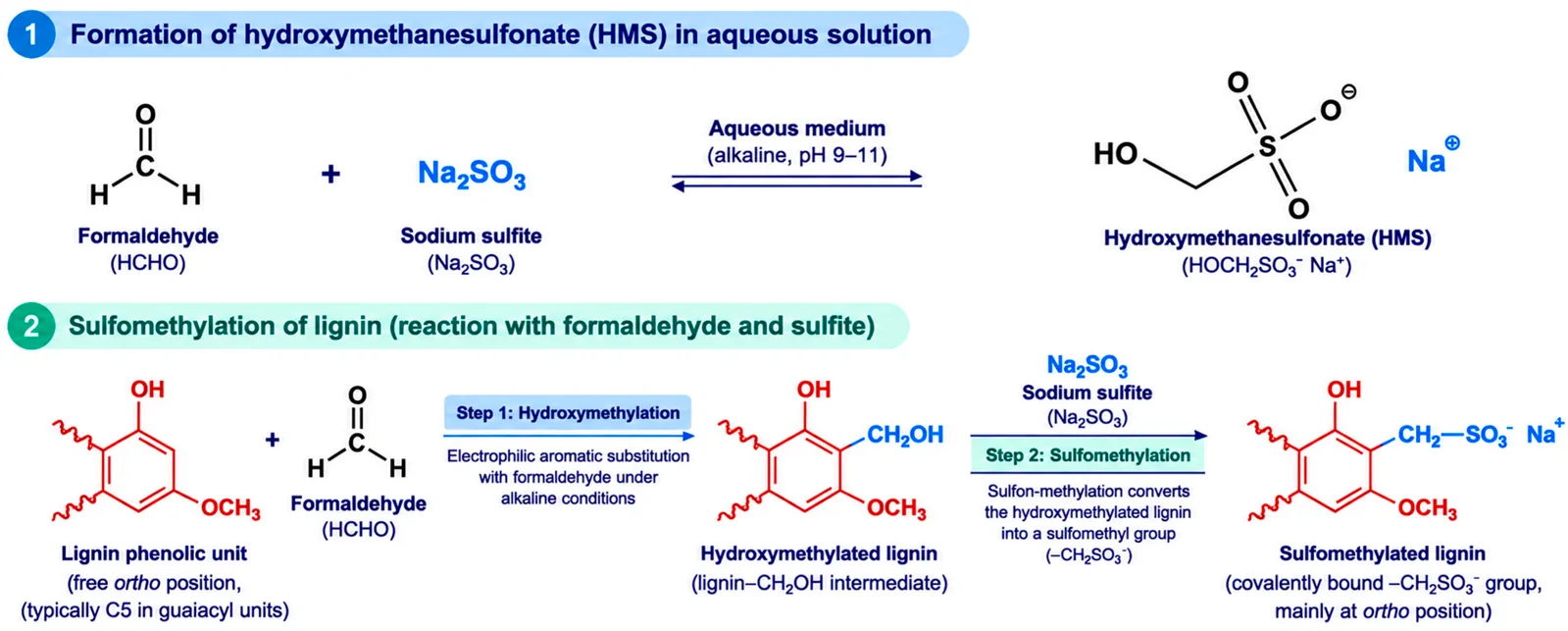
Chemical reaction scheme of lignin sulfomethylation. Hydroxymethylsulfonate (HMS) forms from formaldehyde and sodium sulfite in an alkaline aqueous medium, then reacts with a reactive phenolic lignin site to introduce covalently bound sulfomethyl (–CH_2_SO_3_^−^) groups into the lignin structure.

Sulfomethylation is a promising chemical functionalization method to convert relatively hydrophobic, poorly water-soluble technical lignins into anionic, water-dispersible structures. The sulfonate-containing sulfomethyl groups are added to the lignin structure. The reaction is usually carried out in aqueous alkaline media using formaldehyde and sodium sulfite (Na_2_SO_3_), which first react to form hydroxymethanesulfonate (HMS) species, which then impart functionalities to lignin [75,76,77]. The main chemical steps involved in lignin sulfomethylation are schematically illustrated in Figure 2. Through this process, covalently bound sulfomethyl groups are formed, and the anionic charge in the lignin is increased [75,76,78]. Sulfomethylation is most active at the reactive aromatic positions of phenolic lignin units. However, the sulfomethylation of the lignin is significantly affected by the lignin origin, S/G content, methoxylation, phenolic hydroxyl content, the molecular weight distribution, and the reaction severity [76,79]. Therefore, the addition of –CH_2_SO_3_^−^ groups is expected to increase the hydrophilicity and water solubility of lignin and influence its molecular conformation, surface charge, thermal behavior, and interfacial behavior [56,77,80].

Recent studies on Kraft and Organosolv lignins have confirmed that sulfomethylation effectively reacts with lignin hydroxyl functionalities, generating more polar materials. The extent of modification depends on the chemical structure of the lignin. Notably, sulfomethylated Organosolv lignin exhibits enhanced dispersion, conductivity, and reduced particle size compared to unmodified lignins [76]. The degree of sulfomethylation is a critical parameter because increasing the density of sulfonate groups generally enhances water dispersibility and electrostatic stabilization. However, excessive reaction severity can lead to competing condensation or degradation reactions, which alter the molecular-weight distribution [77,79,81]. Recent studies on sulfomethylated alkali lignin have demonstrated that controlling the sulfonic group content directly affects particle size and dispersion stability, with optimized materials achieving nanoscale particle sizes and performance comparable to that of commercial lignin dispersants [81]. Similarly, sulfomethylated Kraft lignin has been successfully employed as a selective depressant in mineral flotation, where the introduced sulfonate groups increase lignin’s hydrophilicity and surface affinity, enabling selective interactions with mineral surfaces [56,82]. Sulfomethylation has also been shown to modify lignin–protein interactions: sulfomethylated lignins derived from poplar and Masson pine enhanced the enzymatic hydrolysis of lignocellulosic substrates by preferentially interacting with cellulases and reducing their nonproductive adsorption onto residual lignin [83]. More recent studies have extended the use of sulfomethylated lignin to functional polymeric materials, including polyurethane foams, where the degree of sulfonation influences the thermal stability and mechanical properties [84], and to pesticide formulations, where sulfomethylation increases water dispersibility and, in combination with oxidative pretreatment, enhances antioxidant and dispersant performance [85].

Thus, sulfomethylation provides a versatile route to tune the charge, hydrophilicity, colloidal stability, and interfacial reactivity of technical lignin, transforming an otherwise poorly water-soluble macromolecule into a functional polyelectrolyte suitable for use as a dispersant, flocculant, mineral-processing reagent, polymeric material, or other advanced applications.

Sulfomethylation should therefore be distinguished from sulfonation not only on the basis of chemical structure but also in terms of the resulting functional role. Introducing –CH_2_SO_3_^−^ groups substantially increases lignin charge density and water dispersibility, which can facilitate interfacial interactions and processing in aqueous systems. However, increased ionic character does not necessarily translate directly into higher intrinsic charge-storage capacity. Its main value for EES applications may instead arise from improved electrolyte interaction, dispersion, precursor assembly, or interface control in composite electrodes. Consequently, we should evaluate electrochemical benefits based on the specific function of sulfomethylated lignin rather than inferring them solely from its increased sulfonate content.

#### 4.3.3. Esterification

Esterification is a chemical modification strategy that modifies the hydroxyl groups of lignin to optimize its polarity, hydrophobicity, solubility, and processability. Lignin is characterized by phenolic and aliphatic hydroxyl groups, which are different in the lignin source and isolation process. These functionalities are the primary reactive sites for chemical modification, but their high intra- and intermolecular hydrogen bonding is responsible for lignin aggregation and lack of compatibility with nonpolar or weakly polar polymer matrices. Esterification converts these hydroxyl groups to ester functionalities by replacing the hydroxyl groups with the groups O–CO–R, where R is an alkyl, aryl, or other acyl group [57,86]. This can reduce hydrogen bonding within the lignin structure and increase its flexibility, thereby enhancing its organic solubility, hydrophobicity, and thermoplasticity.

Lignin esterification is usually performed using carboxylic acids or their derivatives, such as acid anhydrides and acyl chlorides, in the presence of acid or base catalysts. Acetic anhydride is used to produce lignin acetate, whereas long-chain fatty acid chlorides or anhydrides can form hydrophobic alkyl chains in the lignin structure. The reaction conditions and catalysts influence the extent of modification. For example, acetylation catalyzed with 1-methylimidazole or 4-dimethylaminopyridine (DMAP) provided higher degrees of esterification than sodium acetate or sulfuric acid, while the absence of a catalyst was found to give much lower conversion [86].

The acyl group of the new material determines its properties. In organic solvents, acetylation can improve lignin solubility, while the introduction of long aliphatic chains produces more hydrophobic derivatives and can reduce the glass-transition temperature by weakening intra- and intermolecular hydrogen. Esterified lignin has better thermal and oxidative stability and better flexibility, and it can also be more compatible with nonpolar polymers [57,86]. De Oliveira et al. [87] showed that microwave acetylation of Kraft lignin with acetic acid as the solvent and acetylating reagent with different aliphatic hydroxyl groups could lead to better solubility of Kraft lignin acetates in chloroform, ethyl acetate, and dichloromethane. The modified lignin also exhibited better antioxidant activity, suggesting that selective esterification can improve processability while preserving the functional properties of the aromatic structure [88].

These changes in polarity and interfaces are particularly important for polymer composites and surface treatments. Esterification can increase lignin hydrophobicity and improve its compatibility or miscibility with hydrophobic matrices; for example, oleyl esterification improved lignin incorporation into sulfur-rich composites [89]. Esterification of lignin with palmitoyl chloride improved its compatibility with PLA. Compared with films containing unmodified lignin, the esterified-lignin/PLA films exhibited a smoother and more homogeneous morphology, higher tensile strength, and lower water absorption; however, their tensile properties remained below those of neat PLA [90]. Esterified lignin has also been investigated for coatings, where fatty acid chains add hydrophobicity to the surface, and in lignin-based polymer systems, such as polyesters, elastomeric materials, and epoxy resins [91].

More recent approaches extend esterification beyond solvent-based routes by using microwave heating, reactive extrusion, supercritical CO_2_, and enzymatic processes to reduce reaction time, solvent use, and dependence on conventional reagents. Enzymatic esterification is particularly promising because lipases selectively esterify aliphatic hydroxyl groups under mild conditions while preserving the lignin’s aromatic structure. Martinez-Garcia et al. [92] used Candida antarctica lipase B to esterify a lignin-derived monomer with fatty acids, producing oil-soluble phenolic esters that retained radical-scavenging activity [87,92].

The effect of esterification is therefore strongly dependent on the nature and degree of acylation. Moderate modification can improve compatibility with nonpolar matrices and alter lignin’s thermal and mechanical behavior, whereas extensive incorporation of hydrophobic chains may decrease electrolyte affinity and limit ionic accessibility. Consequently, esterification is better regarded as a tool for controlling interfacial and processing properties than as a universal route to increasing electrochemical activity. We assess its usefulness in EES by whether the modified lignin functions as an active redox component, binder, dispersant, or precursor for subsequent carbonization.

Table 2 summarizes the chemical modification strategies that alter lignin structure and properties for specific applications. Esterification converts phenolic and aliphatic hydroxyl groups into ester functionalities (–O–CO–R), thereby reducing intermolecular hydrogen bonding and increasing hydrophobicity, organic solubility, flexibility, and processability. Carboxymethylation, by contrast, adds carboxymethyl (–CH_2_COO^−^) groups to phenolic and aliphatic hydroxyl groups, thereby increasing the negative charge density, hydrophilicity, and coordination ability of the polymer. Sulfomethylation also introduces sulfomethyl (–CH_2_SO_3_^−^) groups at reactive aromatic sites, increasing the negative charge, water solubility, and dispersibility of lignin. Taken together, these chemical transformations show how targeted functionalization of specific structural sites can tailor lignin’s physicochemical properties and expand its utility across a variety of applications.

## 5. From Lignin to Functional Carbon Materials

**Figure 3:**
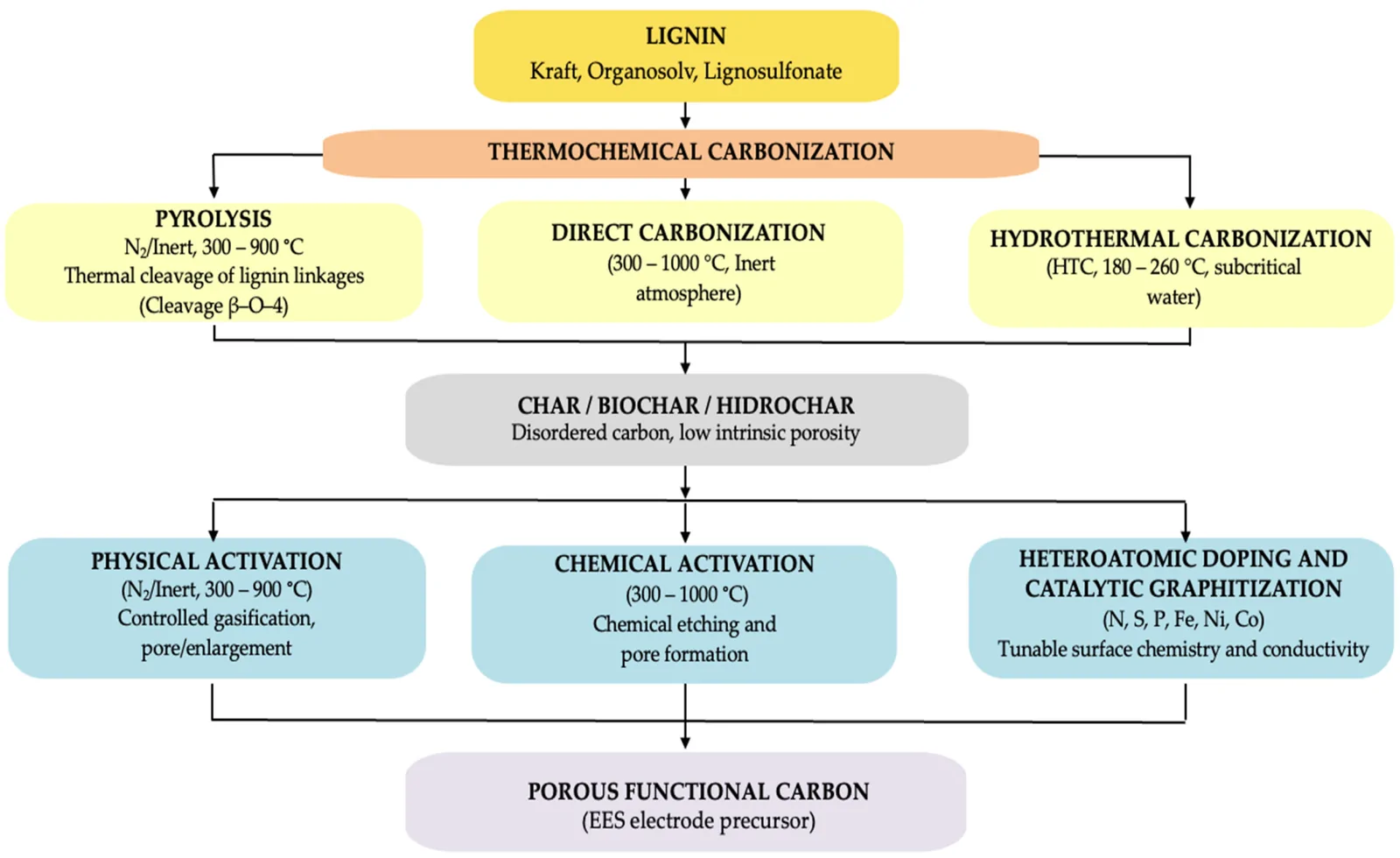
Thermochemical conversion pathways of technical lignins into porous functional carbon materials for electrochemical energy storage (EES).

The polyphenolic macromolecule transforms into a solid, electrically conductive, and structurally stable carbon through three main thermochemical pathways: pyrolysis, direct thermal carbonization, and hydrothermal carbonization (HTC), as summarized schematically in Figure 3. These processes produce an intermediate product (char, biochar, or hydrochar) that is still dense and has low intrinsic porosity; this product must undergo subsequent stages of physical or chemical activation, heteroatom doping, and/or catalytic graphitization to acquire the surface area, hierarchical porosity, and electrical conductivity required for a high-performance electrodes.

### 5.1. Pyrolysis and Direct Carbonization

Pyrolysis involves heating lignin in the absence of oxygen (typically under N_2_ or Ar) at temperatures between 300 and 900 °C, which causes the progressive breakdown of its ether bonds (mainly β–O–4) and carbon–carbon bonds (5–5, β–β, β–5), resulting in an aromatic solid residue (char), a liquid fraction (bio-oil, rich in phenols), and non-condensable gases (CO, CO_2_, CH_4_, H_2_) [93]. This process is generally divided into three distinct stages. The initial stage, which occurs at temperatures below 300 °C, involves drying and the release of light volatiles. The primary stage, spanning 300–500 °C, is characterized by extensive depolymerization, primarily through demethoxylation and homolytic cleavage of ether bonds, resulting in the formation of specific phenolic monomers, such as guaiacol, syringol, and their phenolic derivatives. The final stage, carbonization, occurs at temperatures exceeding 500 °C, where the remaining aromatic structures condense and aromatize, forming the polycyclic carbon matrix that constitutes charcoal, while expelling the last heteroatoms as H_2_O, CO, and CO_2_ [94,95].

The final structure and properties of the char are governed by the combined effects of the maximum carbonization temperature, heating, and residence time, rather than by temperature alone. Increasing the final temperature generally promotes deoxygenation, aromatic condensation, and structural ordering, resulting in lower H/C ratios, fewer oxygen-containing surface groups, and higher electrical conductivity of the char [96]. However, the thermal history also affects the extent and pathway of these transformations. In particular, the heating rate influences volatile release, secondary reactions, and the evolution of surface functionalities. Li et al. [97] showed that increasing the heating rate during lignin pyrolysis altered functional-group composition and promoted gas formation through accelerated cracking reactions, while shorter residence times at high heating rates limited the extent of deoxygenation. More broadly, both heating rate and residence time influence the competition between primary lignin decomposition, secondary vapor-phase reactions, and repolymerization toward char, thereby affecting product yield and composition [98,99]. Consequently, the carbonization temperature should be considered together with the heating rate and residence time when tailoring char properties for subsequent activation or electrochemical applications.

The inorganic composition of the precursor provides an additional factor controlling these transformations. Residual metals from the Kraft pulping process, particularly Na, K, and Ca, can catalyze secondary reactions during thermal treatment, thereby influencing both char yield and structural ordering. At temperatures above approximately 600 °C, condensation and aromatization of the remaining fragments progressively generate turbostratic carbon domains, which can serve as precursors to more ordered graphitic structures during subsequent catalytic graphitization [96]. This structural evolution therefore reflects not only the imposed temperature but also the precursor’s chemical composition and thermal history.

Furthermore, Chen et al. (2021) [100] studied the effect of acidic protons on lignin cleavage pathways, which is relevant in catalytic pyrolysis processes using solid acid catalysts. Using density functional theory (DFT) calculations, these authors showed that protonation of the oxygen atom in the β–O–4 bond reduced the dissociation energy of the Cβ–O bond from 210 to 179 kJ mol^−1^, thereby facilitating its cleavage and promoting the formation of phenol, p-hydroxybenzaldehyde, and p-hydroxyacetophenone as primary products.

### 5.2. Hydrothermal Carbonization (HTC)

Unlike conventional pyrolysis, hydrothermal carbonization (HTC) is particularly attractive for the valorization of wet lignin streams because it operates in subcritical water, typically at 180–280 °C under autogenous pressure, with residence times of approximately 1–8 h, thereby avoiding energy-intensive drying [101]. This feature is especially relevant to industrial Kraft lignin and lignosulfonate streams, where high moisture content can limit the feasibility of conventional thermochemical conversion. Under hydrothermal conditions, the enhanced ionization of water promotes acid- and base-catalyzed reactions involving lignin, including ether-bond hydrolysis, dehydration, decarboxylation, and condensation of soluble phenolic and carbonyl intermediates. These reactions progressively convert dissolved lignin fragments into condensed carbonaceous structures, typically yielding spherical hydrochar particles in the micro- to sub-micrometer range [102,103].

The resulting hydrochar differs structurally from that produced via high-temperature pyrolysis. Wikberg et al. [103], using ^31^P NMR and FTIR spectroscopy, showed that the HTC of Kraft lignin at 200–250 °C decreases methoxyl and aliphatic side-chain functionalities while increasing the relative contribution of condensed aromatic structures. Simultaneously, hydrochars generally retain a higher concentration of oxygen-containing surface groups, including carboxyl, hydroxyl, carbonyl, and phenolic functionalities, than chars produced under more severe thermal conditions [104,105]. This surface chemistry can be advantageous for electrochemical applications because oxygenated groups may contribute to pseudocapacitance and provide reactive sites for subsequent activation or heteroatom functionalization reactions. However, the relatively mild carbonization conditions also lead to limited initial pore development, necessitating subsequent activation or thermal treatment when high surface area and accessible pore volume are required.

The HTC operating conditions strongly influence the balance between the solid yield, energy density, and precursor reactivity. Salcedo-Puerto et al. [106] reported that increasing the temperature from 180 to 260 °C enhanced the energy densification of industrial Kraft lignin, with higher gross calorific values above approximately 220 °C, but reduced the solid yield as decarboxylation became more pronounced. Importantly, the carbonization temperature affects the precursor’s structure and its response to subsequent activation. Rodriguez Correa et al. [107] showed that the severity of the initial carbonization step influences the development of micro- and mesoporosity during activation, highlighting the importance of optimizing HTC conditions according to the properties required for the final carbon material.

Feedstock composition and pretreatment provide additional means of controlling hydrochar formation. Low-molecular-weight lignin fragments may remain in the aqueous phase unless their conversion to the solid fraction is promoted. Kang et al. [108] investigated the HTC of isolated lignin at 225–265 °C and found that lignin produced higher hydrochar yields than wood meal, cellulose, and D-xylose under comparable conditions. The lignin-derived hydrochars exhibited aromatic and phenolic structures, carbon contents of 63–75%, and higher heating values of 24–30 MJ kg^−1^. These results highlight the inherent tendency of lignin to retain carbon in the solid phase during HTC.

HTC can also be integrated with subsequent thermochemical conversion processes. For instance, HTC followed by slow pyrolysis enables a sequential increase in thermal severity and generates carbon materials with more developed, potentially hierarchical porosity from lignin-rich residues [109]. Accordingly, HTC should be regarded as a complementary route to pyrolysis, particularly for processing wet lignin streams. Its main advantage lies in the direct conversion of these streams into oxygen-functionalized carbonaceous precursors at relatively low temperatures, which can subsequently be tailored through activation, pyrolysis, or chemical functionalization for applications such as electrochemical energy storage.

### 5.3. Physical and Chemical Activation: Porosity Generation

Pyrolytic char and hydrochar generally exhibit relatively low Brunauer–Emmett–Teller (BET) specific surface areas of less than 10–50 m^2^ g^−1^, which are insufficient for electrochemical applications in which capacity depends on the formation of an electric double layer. Therefore, these precursors must undergo an activation step to develop a porous structure accessible to the electrolyte [110].

Physical activation is based on the partial gasification of carbon using CO_2_ or water vapor, typically at 700–900 °C. The gasifying agents preferentially react with sites of higher reactivity, such as defects, aromatic edges, and amorphous carbon, progressively generating micro- and mesopores. The main reactions are the Boudouard reaction (C + CO_2_ → 2CO) and steam gasification (C + H_2_O → CO + H_2_). This process offers operational and environmental advantages by eliminating the need for corrosive chemicals and reducing wash effluent generation. However, it typically yields smaller surface areas than those achieved by chemical activation [111,112].

Chemical activation uses agents such as KOH, NaOH, ZnCl_2_, H_3_PO_4_, and K_2_CO_3_, typically by impregnating the precursor and then heat-treating it. Among these, KOH stands out for its ability to produce high microporosity and a high specific surface area. During treatment, KOH reacts with the carbonaceous matrix through a sequence of transformations involving K_2_CO_3_, K_2_O, and metallic-K. These species promote the gasification of carbon and the intercalation of potassium into the aromatic domains, and their subsequent removal during washing creates a pore network in the carbon matrix [113]. The intensity of this process depends heavily on KOH concentration and activation temperature. Wang et al. [114] reported surface areas of up to 2842 m^2^ g^−1^ for hierarchically porous lignin-derived carbon, while Pan et al. [115] obtained a surface area of 2285 m^2^ g^−1^ and a specific capacitance of 291.3 F/g under KOH activation conditions. However, excess KOH can intensify gasification, reduce the carbon yield, and cause excessive pore development or widening.

Activation with H_3_PO_4_ occurs at lower temperatures, typically between 400 and 600 °C, through dehydration, cross-linking, and the transient formation of phosphate species. During treatment, phosphate-ester bonds and polyphosphate species promote matrix stabilization and limit shrinkage; their subsequent removal results in a structure with a higher proportion of mesopores. This characteristic allows the production of materials with more open and hierarchical porosity, as well as surface areas exceeding 2000 m^2^ g^−1^ in some lignin-derived carbons [116]. This method can also reduce thermal stress compared to alkaline activation, although it requires subsequent washing to remove phosphate species [117,118].

A comparison of different strategies confirmed that the activating agent and temperature jointly determine the porous architecture and surface chemistry of carbon. Peñas-Garzón et al. [119] observed that KOH promotes a high surface area and microporosity, whereas H_3_PO_4_ produces a broader pore distribution. Similarly, Bergna et al. [120] demonstrated that the choice between chemical and physical activation modifies both the micro-/mesopore ratio and the concentration of surface functional groups..

In the context of electrochemical energy storage, the selection of the activation method must therefore consider not only the BET surface area, but also the pore size distribution and surface chemistry. KOH is particularly suitable for maximizing microporosity and specific surface area, whereas H_3_PO_4_ may be preferable for developing a more open structure with a greater mesoporous contribution. Physical activation is a simpler and more environmentally friendly alternative when extreme surface development is not required.

**Figure 4:**
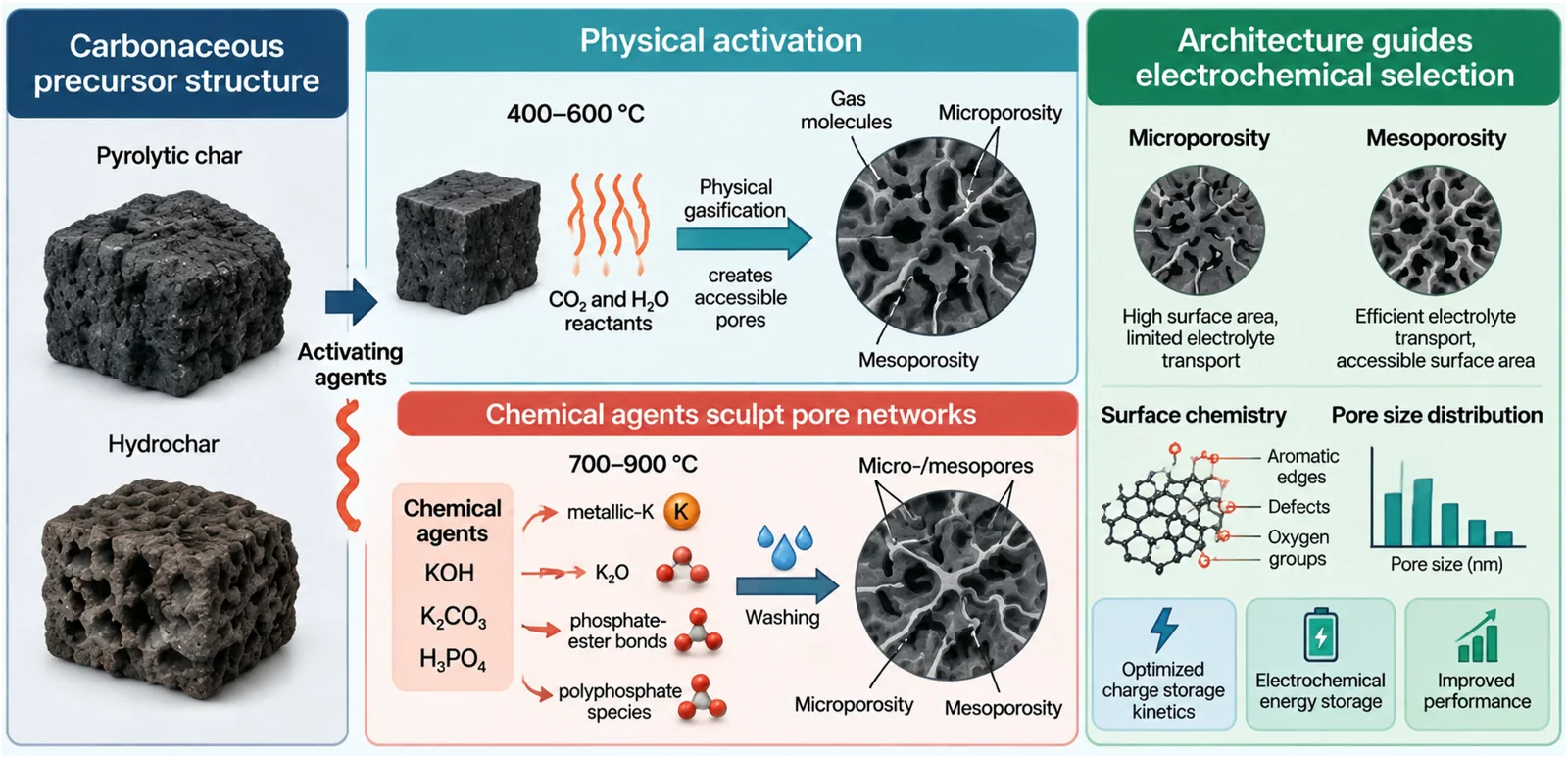
Activation strategy governs porous carbon architecture.

These relationships are summarized in Figure 4 (during the preparation of this manuscript, we used ChatGPT (OpenAI, GPT-4) to assist in the creation of graphic elements for Figure 4).

### 5.4. Heteroatom Doping and Catalytic Graphitization

Beyond pore development, the electrochemical properties of lignin-derived carbons can be tailored by heteroatom doping, particularly with N, S, and P. These elements modify the electronic structure and surface chemistry of the carbon matrix, introducing redox-active sites, increasing electrolyte wettability, and improving electronic transport, depending on their configuration [121]. Heteroatoms can be introduced intrinsically from the lignin precursor or, more commonly, through co-carbonization with N-, S-, or P-containing compounds, such as urea, melamine, or thiourea.

Nitrogen doping is particularly effective because N can be incorporated into the carbon framework in pyridinic, pyrrolic, graphitic, and pyridinic-N-oxide configurations, each of which influences the material differently. Pyridinic and pyrrolic N are mainly associated with edge sites and structural defects and can contribute to surface redox reactions, whereas graphitic N replaces carbon atoms within the aromatic lattice and primarily modifies the electronic conductivity [122]. Zhao et al. [123]. reported an N,S-co-doped lignin-derived carbon containing up to 21.6 at. %N, achieving 419 mAh g^−1^ with 96.6% capacity retention after 1000 cycles in a hybrid device. Similarly, N-doped microporous carbons derived from Kraft lignin, with approximately 4–5 at. %N and BET areas near 1000 m^2^ g^−1^, exhibited substantially improved capacitance compared with their undoped counterparts.

Sulfur doping introduces S-containing functionalities, including thiophenic species, which can provide additional redox-active sites and modify the interaction between the carbon surface and the electrolyte. More importantly, the combination of N and S can provide complementary electronic effects and improve the electrochemical responses. For example, Fu et al. [124] obtained hierarchically porous N,S-co-doped lignin-derived carbon with a BET surface area of 2538.3 m^2^ g^−1^ and a specific capacitance of 393 F/g, highlighting the combined contribution of hierarchical porosity and heteroatom-derived active sites [124]. Ionic liquids have also been explored as carbonization media and structure-directing agents, with their chemical nature influencing both pore development and heteroatom incorporation [125]. Although less extensively studied than N and S doping, phosphorus doping offers an additional route to modify surface polarity, charge distribution, and redox behavior.

The effects of heteroatom incorporation extend beyond charge storage. Lignin-derived heteroatom-doped carbons have also demonstrated catalytic activity in electrochemical reactions. For example, N-containing porous carbons have been investigated as oxygen-reduction catalysts in Zn–air batteries [126]. Thus, the choice of dopant and its chemical configuration should be guided by the targeted electrochemical process, rather than simply maximizing the total heteroatom content.

A complementary strategy is catalytic graphitization, which targets structural rather than primarily chemical modification of carbon. Transition-metal species such as Fe, Ni, Co, and Cu catalyze the transformation of disordered carbon into more ordered graphitic domains via dissolution–precipitation mechanisms, typically at 900–1100 °C. Yan et al. [127] compared Fe, Ni, Co, and Cu in the catalytic graphitization of Kraft lignin and showed, by Raman spectroscopy and HRTEM, that Fe and Ni were particularly effective in generating few-layer graphene-like structures. The resulting increase in structural order and electrical conductivity can reduce the internal resistance of carbon electrodes, making catalytic graphitization particularly relevant for high-power EES applications such as lithium-ion batteries.

Overall, heteroatom doping and catalytic graphitization address the complementary limitations of lignin-derived carbons: doping tunes the surface chemistry and electronic structure, whereas graphitization improves the structural order and charge transport. Therefore, their combination with controlled activation provides a versatile strategy for independently adjusting porosity, surface reactivity, and conductivity to meet the requirements of the targeted energy-storage device.

The structure of lignin carbon can be further tuned through physical and chemical activation, heteroatom doping, and catalytic graphitization following carbonization. As shown in Table 3, these strategies modify the carbon structure in different ways. For example, physical activation selectively gasifies carbon at edges and defects, whereas chemical activation promotes the formation of microporous and mesoporous networks using activation agents such as KOH and H_3_PO_4_. In addition, heteroatoms such as N, S, and O can be incorporated into the carbon layer, forming new redox-active and polar surface sites that enhance electrolyte affinity and contribute pseudocapacitively. In addition, catalytic graphitization of the carbon layer with transition metals will lead to more ordered graphitic regions and better electronic conductivity. It can be used to balance the surface area, pore structure, surface chemistry, and electrical conductivity depending on the application, for instance, in electrochemical energy storage.

Among the strategies discussed in Section 5.1, Section 5.2, Section 5.3 and Section 5.4, direct pyrolysis followed by controlled chemical activation represents the most convenient route for producing lignin-derived carbons for electrochemical energy storage. Pyrolysis provides a robust and scalable carbonization step, while chemical activation, particularly with KOH, efficiently generates the high surface area and interconnected microporous/mesoporous structure required for electrolyte-ion accessibility and electric double-layer charge storage. This combination consistently produced lignin-derived carbons with high surface areas and competitive capacitance; however, activation severity must be optimized because excessive KOH loading can reduce carbon yield, widen pores excessively, and increase processing demands for chemical recovery and washing. Hydrothermal carbonization is a more attractive initial route when wet Kraft lignin or lignosulfonate streams are available, as it avoids energy-intensive drying; however, it generally requires a subsequent activation or pyrolysis step to achieve adequate conductivity and pore development. Heteroatom doping can further enhance wettability and pseudocapacitive contributions, whereas catalytic graphitization is particularly relevant for battery anodes and high-power devices that require improved electrical conductivity and structural order. Therefore, a pyrolysis–activation route should be the preferred baseline for supercapacitor-grade lignin carbon, while HTC, heteroatom doping, and catalytic graphitization should be selected as complementary modifications based on feedstock moisture, target pore architecture, and the final energy-storage application [96,106,121,123,126,130].

### 5.5. Relationship Between Porous Structure and Electrochemical Performance

The relationship between the surface area and electrochemical performance of porous carbons is more complex than traditionally assumed. Although a larger BET surface area can increase the number of sites available for electric double-layer formation, capacitance does not necessarily scale linearly with this parameter. Abouelamaiem et al. [131] demonstrated, through three-dimensional reconstruction of the nanostructure of biochar using X-ray tomography, that the electrochemical performance also depends on the connectivity of the porous network and the accessibility of ions, which determine the fraction of surface area that is usable during charging and discharging. In a hierarchical architecture, macropores facilitate electrolyte access, mesopores promote inward transport, and micropores provide a large portion of the active surface area for charge storage. Therefore, their contributions must be evaluated in an integrated manner, not solely based on the BET surface area.

This limitation of the approach, which is based exclusively on porosity, becomes even more evident when considering the electronic structure and degree of disorder in carbon. Liu et al. [132] combined in situ NMR, X-ray scattering, and molecular simulations to show that the size, orientation, and curvature of local graphene domains can predict capacitance better than pore size considered in isolation [132]. Thus, carbon materials with similar BET surface areas and pore distributions may exhibit different electrochemical responses owing to variations in their local structures. This aspect is particularly relevant for lignin-derived carbons, whose heterogeneous composition and reactivity favor the formation of aromatic domains with varying degrees of condensation and disordering.

The nature of the electrolyte introduces another variable. The optimal micropore-to-mesopore ratio depends on the size, solvation, and mobility of the ionic species. Micropores can provide a high density of storage sites when they are accessible to the electrolyte. In contrast, a greater mesoporous contribution is favorable when the transport of bulky or multivalent species limits the charging kinetics [133,134]. In this regard, Pan et al. [115]. observed in KOH-activated lignin carbons that moderate activation conditions, around 800 °C, provide a favorable compromise between the development of microporosity and the preservation of a red mesopore capable of sustaining ion transport at high current densities [115].

Consequently, the design of lignin-derived carbons for electrochemical storage must shift from a strategy focused on maximizing BET surface area to one that engineers the ionic accessibility, pore connectivity, and electronic structure of the carbon. These characteristics can be adjusted by controlling carbonization temperature, activation conditions, and heteroatom doping, thereby tailoring the architecture and surface chemistry to the target electrolyte and device [135,136]. This structure–transport–storage relationship constitutes a mechanistic link between lignin transformation stages and the material’s final performance in energy storage devices.

## 6. Electrochemical Energy Storage: Fundamentals and Lignin-Based Materials for EES Applications

### 6.1. Fundamentals of Electrochemical Energy Storage (EES) Devices

Electrochemical energy storage (EES) devices store and release electrical energy through electrochemical processes. Batteries primarily rely on oxidation–reduction reactions, whereas electrochemical capacitors store charge through electrostatic ion adsorption and/or rapid Faradaic reactions. During discharge, the current represents the rate of charge transfer through the external circuit and depends on the external load, reaction kinetics, and internal resistance [137,138,139]. These storage mechanisms enable electrochemical devices to serve a wide range of applications, from portable electronics to grid-scale energy storage, supporting the development of sustainable energy systems [140,141,142,143].

According to the energy storage principle, EES devices are commonly classified as batteries, electrochemical capacitors (supercapacitors), and hybrid battery–capacitor systems. Batteries are typically characterized by their high energy density and consistent operating voltage. They are generally classified into primary and secondary (or rechargeable) batteries [141]. Electrochemical capacitors are characterized by their high power density and longer cycle life but lower energy density than batteries. They are commonly divided into electrochemical double-layer capacitors (EDLCs), which store charge through reversible electrostatic ion adsorption at the electrode–electrolyte interface, and pseudocapacitors, which additionally store charge through rapid, reversible Faradaic reactions at or near the electrode surface [144,145,146]. EES devices are manufactured in units or modules that offer flexible combinations to meet the demands of high energy capacity, fast charge–discharge rates, increased safety, and long life [143].

Batteries consist of one or more electrochemical cells. If the cells are connected in series, the battery is designed to provide a specific voltage (Vd), whereas if they are connected in parallel, they provide a specific current (Id). The energy density of a cell depends on the total charge transferred (per weight or volume) for full discharge in a period and at a specific and constant current. This is called the specific capacity (Q(Id)) and is an important factor to consider when designing a battery [147,148]. It is desirable to design batteries with the fewest number of cells because they require simpler management and lower costs. Therefore, cells with high energy density are of great interest.

A typical battery cell contains two electrodes, an electrolyte, and a separator. During discharge, oxidation occurs at the anode and reduction at the cathode. Electrons flow through the current collectors and external circuit, whereas ions migrate through the electrolyte to maintain electroneutrality; thus, the electrolyte must provide ionic conductivity while limiting electronic conduction between electrodes. The separator prevents direct electrical contact and short circuits while allowing ionic transport [149,150].

Lithium-ion batteries (LIBs) are among the most important and widely used electrochemical energy storage technologies today. Electrodes are interfaces where lithium-ion intercalation and de-intercalation reactions occur, and the electrode architecture and morphology are essential for optimizing ion and electron transport. Nanoporous structures and nanocomposite-based materials can increase the reaction surface area and improve the kinetics of the electrochemical reactions [151,152,153]. Cathode and anode materials are critical for the performance of lithium-ion batteries. Typical cathodes include compounds such as LiCoO_2_, LiFePO_4_, and LiNiMnCoO_2_, whereas anodes are commonly made of graphite. In accordance with the principle and purpose of EES devices, the electrolyte must generally meet the following requirements: (1) wide electrochemical window; (2) high ionic conductivity; (3) high chemical and thermal stability; (4) chemical inertness to other cell components, such as the separator, electrode substrates, and cell packaging materials; and (5) safety, nontoxicity, and economic affordability [154].

Supercapacitors (SCs), along with LIBs, are among the most promising devices for energy transitions. Like batteries, they consist of two electrodes (anode and cathode), a separator, and an electrolyte. They store and release energy by adsorbing or desorbing ions at the interface between the electrode materials and the electrolyte, with the difference that most SCs use carbon pores or electrodes with larger surface areas and thinner dielectrics to collect a large number of charges. This type of system offers numerous advantages, such as high capacitance, long cycle life, low internal resistance, fast charge and discharge rates, non-destructive materials, lower cost per cycle, and high efficiency per cycle (up to 95%) [155,156].

### 6.2. Derived Carbons for Supercapacitors (EDLCs and Pseudocapacitors)

Supercapacitors utilizing lignin-derived carbon represent the most advanced and extensively investigated application of this biopolymer in electrochemical energy storage systems. Their attractive performance stems from their unique ability to combine the high surface area and hierarchical porosity generated through activation processes with additional faradaic contributions from residual oxygen-containing functional groups, particularly quinone/hydroquinone redox couples. Therefore, the electrochemical behavior of lignin-derived carbons is governed not only by the accessible surface area but also by pore architecture, surface chemistry, and electrolyte ion transport.

Among processing strategies, chemical activation has been widely used to enhance pore development and increase the number of electrochemically accessible sites. Pan et al. [115] synthesized activated carbon from enzymatically hydrolyzed lignin through a single-step KOH activation process, producing an interconnected porous structure with a surface area of 2285 m^2^/g. The optimized electrode (LAC800-4) achieved a specific capacitance of 291.3 F/g in a three-electrode configuration and 186.8 F/g in a symmetric supercapacitor, while maintaining 96.1% capacitance retention after 10,000 cycles at 20 A/g and delivering an energy density of 6.5 Wh/kg [115]. These results highlight that high surface area contributes to charge storage; however, the decrease in capacitance when transitioning from a three-electrode system to a complete symmetric device also demonstrates that BET surface area alone cannot predict practical device performance.

Beyond pore generation, surface functionalization through heteroatom incorporation has emerged as an effective strategy to improve the electrochemical response of lignin-derived carbons. Nitrogen and sulfur co-doping, achieved either from sulfur-containing lignin precursors such as lignosulfonate or through external nitrogen/sulfur sources, can enhance surface polarity, electrolyte wettability, and additional faradaic interactions. Fu et al. [124] combined lignin micelles with sulfomethylated melamine using a magnesium carbonate template, obtaining a hierarchical porous carbon with a surface area of 2538.3 m^2^/g and a gravimetric capacitance of 393 F/g at 0.5 A/g. Similarly, Chen et al. [157] prepared N/S-containing carbon through the self-assembly of lignosulfonate with Fe^3+^ ions without chemical activation or external templates. The resulting material contained 8.47% nitrogen and 2.56% sulfur, achieving 390 F/g and an energy density of 14.7 Wh/kg. These studies indicate that precursor chemistry and surface functionalization can provide comparable or superior electrochemical performance without relying exclusively on maximizing surface area.

Beyond activation and heteroatom engineering, other approaches have focused on exploiting lignin’s intrinsic chemical functionality and structural versatility. A recent review dedicated to lignin-derived electrodes for supercapacitors highlighted the relationship between lignin precursor characteristics, processing methods, and capacitive behavior [4]. Zhou et al. [158] investigated the direct contribution of lignin redox activity without complete carbonization by depositing Kraft lignin onto HNO_3_-treated activated carbon. The resulting electrode achieved 293 F/g at 1 A/g, which was attributed to reversible quinone-group reactions. Similarly, Li et al. [159] incorporated lignosulfonate into graphene hydrogels to fabricate flexible, metal-free supercapacitors, demonstrating the potential of lignin-derived functional groups in hybrid carbon architectures. Furthermore, solvent-based soft-templating enabled the direct synthesis of mesoporous carbon fibers from electrospun lignin nanofibers using PVA as a sacrificial polymer. Ago et al. [160] showed that mesoporosity and ion accessibility can matter more than total surface area alone, particularly for flexible electrodes that require rapid ion transport.

The diversity of lignin-derived carbon architectures shows that electrochemical performance results from interactions among precursor chemistry, activation conditions, pore structure, and surface functionality. Table 4 summarizes this relationship by correlating lignin precursor type, carbonization/activation strategy, resulting structural characteristics, and electrochemical response. The comparison highlights that different processing routes can achieve similar capacitance values through distinct mechanisms, including micropore development, mesopore formation, heteroatom incorporation, or preservation of redox-active functionalities.

For comparison, lignin-derived carbon electrodes can also be compared with activated carbons from other biomass residues, but these comparisons must account for large differences in cell configuration, electrolyte composition, activation chemistry, and reporting methodology. Activated carbon derived from Australian industrial hemp hurd exhibited a specific capacitance of 240 F/g at 1 A/g as a single electrode, while the corresponding hybrid sodium-ion capacitor assembled with electrolytic manganese dioxide delivered 105 F/g at the same current density, together with an energy density of 38 Wh/kg at 761 W/kg [161]. Honeydew-peel-derived activated carbon reached higher electrode-level capacitance values of 486 F/g at 0.6 A/g and 478 F/g at 1.3 A/g in 1 M H_2_SO_4_ [162], whereas mango-seed-husk-derived activated carbon achieved 135 F/g at 5 mA cm^−2^ [163]. These values should not therefore be used as direct ranking of biomass precursors since electrochemical performance is dependent on precursor properties.

A common conclusion in all of the studies reported is that no structural descriptor (e.g., BET surface area) can explain the performance of lignin-derived carbons: KOH activation leads to large micropores and high surface areas, but templating strategies, heteroatom incorporation and precursor-assisted self-assembly also affect pore connectivity, electrolyte accessibility and surface chemistry. Since these modifications are often combined in one synthesis route, it is not possible to separate the contribution from each factor individually. Thus, reliable structure–performance relationships require pore size distribution, pore connectivity, surface functionality, electrolyte accessibility, and testing configuration, as well as maximum surface area or specific capacitance values.

### 6.3. Lignin-Based Materials for Lithium-Ion Batteries (Anodes and Cathodes)

Lignin is used as a precursor for hard carbon anodes in lithium-ion batteries because it has a cross-linked aromatic structure and, upon carbonizing, it forms a disordered matrix with Li^+^ insertion sites that are beyond those in regular graphite. Sun et al. [164] fabricated carbon nanofibers from rice straw lignin via electrospinning and heat treatment, yielding an anode with an initial discharge capacity of 1108.3 mAh/g and a stable capacity of 377.3 mAh/g, retaining 84.6% of its capacity after 100 cycles at 1C. Li et al. [165] developed a porous carbon by treating lignin with chloroacetic acid to coordinate metal ions as a self-assembled template and achieved 500 mAh/g at 50 mA/g, retaining 99% capacity after 1000 cycles at 1000 mA/g, which is one of the most stable performances for biomass-based carbon anodes. Similarly, in Li et al. [166], porous carbon was engineered from natural lignin, achieving a specific capacity of 420 mAh/g and 99% retention over 300 cycles at 0.2 A/g [167]. The implementation of structured mesoporosity via a double-template synthesis has also produced impressive results. Huang et al. produced a honeycomb-like mesoporous carbon compound from lignin and SiO_2_ with a pore volume of 2.23 cm^3^/g. This compound also had a reversible capacity of 1109 mAh/g due to the Li^+^ diffusion kinetics produced by the three-dimensional mesoporous structure [168]. Lignin-derived carbon can be combined with electroactive metal oxides to form composites anodes for lithium-ion batteries. Li et al. [166] deposited MnO_2_ nanoparticles on hollow carbon nanospheres made from lignosulfonate. The resulting composite anode delivered a specific discharge capacity of 478 mAh/g after 500 cycles, with a degradation rate of only 0.026% per cycle. These findings illustrate the potential of lignin-derived carbon as a framework for electroactive components in composite anodes. Although the reported capacity exceeds the theoretical capacity of graphite (~372 mAh/g), this comparison must account for the contribution of MnO_2_ to lithium storage.

Although the above studies demonstrate the potential of lignin-derived carbons, their reported capacities should be interpreted against commercial lithium-ion battery benchmarks and equivalent cell configurations. Graphite remains the industrial reference anode because of its low lithiation potential, structural stability, and theoretical capacity of 372 mAh/g, corresponding to the fully lithiated phase LiC_6_ [169]. In practical graphite/PVDF electrodes, reversible capacities around 350 mAh/g after 50 cycles have been reported. As summarized in Table 5, the stabilized capacity of lignin-derived carbon nanofibers (377.3 mAh/g) is therefore comparable with graphite, whereas the 420–500 mAh/g values reported for porous lignin-derived carbons exceed this benchmark [164,165,167]. Nevertheless, capacities close to 1108–1109 mAh/g should be interpreted carefully because they may include irreversible lithium consumption, SEI formation, surface storage, and in the case of the lignin-carbon/SiO_2_ material, the contribution of the SiO_2_-containing composite rather than carbon alone. This distinction is essential because commercial viability also depends on electrode formulation: PVDF binder content can affect electronic contact, porosity, and interfacial resistance, whereas conventional PE, PP, or PP/PE/PP separators influence electrolyte uptake, ionic resistance, thermal stability, and safety.

The wide range of reported capacities also illustrates the difficulty of comparing lignin-derived hard carbons solely on the basis of their maximum specific capacity. Initial discharge capacity, reversible capacity, initial Coulombic efficiency, current density, electrode loading, cycle number, and testing protocol must be considered simultaneously. In addition, lignin precursor chemistry and carbonization conditions jointly determine interlayer spacing, defect density, pore structure, and surface functionality, making it difficult to assign capacity differences to a single processing variable. High initial capacities may therefore coexist with substantial irreversible storage associated with surface reactions or electrolyte decomposition. From a practical perspective, reversible capacity, initial Coulombic efficiency, electrode density, rate capability, and full-cell performance provide more informative indicators than isolated maximum capacity values.

### 6.4. Lignin in Sodium-Ion and Zinc-Ion Batteries: From Anode to Electrolyte Additive

Lignin is a promising feedstock for developing novel energy storage systems, owing not only to its redox properties but also to its heteroatom-rich structure. In this context, sustainability and cost-effectiveness are crucial design considerations. In sodium-ion batteries (SIBs), Mushtaq et al. [173] developed three-dimensional carbon nanofibers from lignin/PLA via electrospinning. The resulting anode delivered a reversible capacity of 330 mAh/g at 100 mA/g in the first cycle and maintained a stable capacity of up to 200 mAh/g over more than 200 cycles [173]. Muddasar et al. [174] synthesized hierarchical porous carbons from lignin (LSPC) via droplet injection using a PVA sacrificial template, achieving surface areas of 426.6–790.5 m^2^/g. The optimized material showed promising performance as an SIB anode, with a reversible capacity of 110 mAh/g at 100 mA/g and a high coulombic efficiency. She et al. [175] showed that natural, uncharred lignin can be directly employed as a high-temperature SIB anode. At 60 °C, additional C=O reactive sites form through homogeneous cleavage of the Cβ–O bond, enhancing performance compared with the results at room temperature [175].

Quaternized Kraft lignin has been identified as a potent electrolyte additive in aqueous zinc-ion batteries (AZIBs), effectively mitigating the conventional challenges of dendrite formation, corrosion, and hydrogen evolution at the zinc anode. Xie et al. (2025) enhanced hardwood kraft lignin by incorporating quaternary ammonium groups (QHKL) and employing it as a 1% by weight additive. This modification notably reduced hydrogen evolution and zinc self-corrosion, thereby facilitating uniform Zn^2+^ deposition. As a result, the Zn//MnO_2_ battery utilizing this electrolyte sustained a specific discharge capacity of 55.4 mAh/g after 3000 cycles at 1.5 A/g, which was approximately 2.5 times greater than that of the reference electrolyte [32]. Similarly, Xu et al. [176] observed comparable improvements in the electrochemical performance using cationic kraft lignin as an additive in aqueous rechargeable zinc-ion batteries.

Furthermore, Memon et al. [177] combined quaternized Kraft lignin (QKL) with polyethylene glycol (PEG200) as co-additives, achieving a coulombic efficiency of 98.01% in Zn//Cu cells and a discharge capacity of approximately 85 mAh/g after 2000 cycles at 1.5 A/g in complete Zn//MnO_2_ cells, with minimal dendritic Zn deposition confirmed by XRD and SEM. These findings indicate that the quaternization of kraft lignin introduces cationic functionalities that regulate the Zn^2+^ interfacial environment, suppress inefficient reactions, and promote uniform Zn deposition. This underscores the potential of chemically tailored lignin as a multifunctional electrolyte additive to enhance the longevity of aqueous zinc-ion batteries.

### 6.5. Lignin as a Separator and Membrane

Commercial polypropylene/polyethylene separators are low-polarity and poorly wettable by organic electrolytes, thereby limiting ionic conductivity and reducing the cell’s thermal safety. The incorporation of lignin, which is rich in polar hydroxyl groups, into matrices systematically improves these properties. Song et al. (2023) developed a polyimide/lignin (PI-L) nanofibrous membrane via electrospinning, which showed a greater affinity for the electrolyte and an ionic conductivity of 1.78 × 10^−3^ S/cm, with a Li^+^ transfer number of 0.787. The LiFePO_4_|PI-L|Li cell retained 95.1% of its capacity after 100 cycles at 1C, outperforming the 90% capacity retention of a commercial polypropylene separator [178]. Yerkinbekova et al. [179]. prepared a photochemically cross-linked electrospun separator made of maleated lignin and polyacrylonitrile (PAN), achieving an ionic conductivity of 2.79 × 10^−3^ S/cm, significantly higher than that of the liquid electrolyte with a commercial Celgard separator (6.5 × 10^−4^ S/cm), and an initial discharge capacity of 147 mAh/g at 0.1C, while also effectively limiting the formation of lithium dendrites for more than 1000 h.

In electrospun solid polymer electrolytes, Coviello et al. [180] combined lignin recovered from kraft liquor by acid precipitation with poly(ethylene oxide) (PEO), showing that incorporating lignin helps counteract dendrite growth and preserve membrane integrity. In Li-S battery fabrication, researchers have used the electron-donating groups on lignin nanoparticles to reinforce Celgard membranes, aiming to induce chemical interactions that retain polysulfides and improve cycling stability. Using scanning electron microscopy (SEM), Zhang et al. [181] observed the presence of macropores and mesopores in Celgard separators, which facilitated polysulfide loss. Coating separators with lignin nanoparticles enables the retention of polysulfides via chemical bonding, thereby enhancing battery stability and cycle life.

### 6.6. Lignin as a Binder and Polymeric Electrolyte

Poly(vinylidene fluoride) (PVDF) is currently widely used as a binder for the manufacture of rechargeable battery electrodes as it has chemical and electrochemical stability but is commonly processed with organic solutions such as N-methyl-2-pyrrolidone (NMP), N,N-dimethylformamide (DMF), or N,N-dimethylacetamide (DMAc) and has negative effects on the ecology, solvent recovery, and complexity of industrial-scale processing. In this context, bio-based binders have been getting more attention as sustainable alternatives.

One of the first demonstrations of this implemented application was given by Lu et al. [182], who used lignin as one binder for the LiFePO_4_ cathode and graphite anode of an LIB. After the electrodes pretreatment that removed the low-molecular-weight lignin fractions‚ the specific capacity was about 117 and 160 mAh/g at the 1C rate for the cathodes and anodes‚ respectively‚ and a low ohmic resistance was also found․ This study shows that the equivalent electrochemical performance can be achieved by smartly controlling the lignin composition and molecular characteristics‚ and that lignin is an efficient material for electrochemical electrodes [182].

Subsequently, the concept of lignin as an electrochemical component was later extended to lithium-metal battery systems. Jeong et al. [183] developed a solid polymer electrolyte based on lignin, functionalized by grafting polymer chains via atom transfer radical polymerization (ATRP). The materials can be cross-linked by UV irradiation to form an attractive polymer network that is capable of maintaining mechanical stability even at high temperatures. This architecture ensured interface stability with metallic lithium and prevented dendrite growth, which is better than that of a reference cell using a liquid electrolyte and a conventional Celgard separator. Lignin is not a typical binder, but it is important to demonstrate that its chemical structure can be modified to produce polymeric materials that play critical roles in stabilizing electrochemical interfaces.

Similarly, lignin’s potential as a precursor for functional carbon materials has enabled its use in energy storage systems beyond conventional batteries. Wang et al. [184] have developed lignin-derived carbon nanofibers in an aqueous system and used them as electrodes in electric double-layer capacitors (EDLCs) by electrospinning the lignin nanofibers to develop them into electrodes with a specific capacitance of 217.2 F/g at 0.2 A/g and a retention of 94.9% after 10,000 cycles. By processing and carbonizing lignin into conductive structures that can change both structurally and electrochemically within the electrode, lignin can be used chemically in a cell or device to increase its conductivity. Its structure is controlled in a way that is useful for the storage process, in which the active materials have significant structural and volumetric changes with cycling.

The same strategy can be applied to Na-ion batteries, as recent research has shown that hard carbon anodes using a lignin-derived binder are economically competitive with those using synthetic binders, offering sufficient capacity and stability during cycling [185]. Since Na-ion batteries need electrodes and interfaces for Na^+^ transport and insertion, these results are very promising. Taken together, the evidence suggests that lignin can be transformed from a passive binder into an active molecule for the design of functional components in electrochemical storage devices.

## 7. Structure–Processing–Performance Relationships: A Critical Analysis

The electrochemical performance of lignin-derived activated carbon is governed by a coupled relationship between the chemical characteristics of the lignin precursor, the processing conditions used during carbonization and activation, and the resulting carbon structure. Consequently, properties such as the BET surface area, pore volume, heteroatom content, degree of graphitization, and surface functionality should not be considered independent descriptors. Rather, they emerge from interconnected transformations occurring throughout thermal conversion and collectively determine ion accessibility, charge-storage mechanism, electronic transport, and cycling stability. Recent studies increasingly support this integrated perspective, moving beyond the conventional assumption that maximizing surface area is sufficient to optimize supercapacitor performance [4,159].

### 7.1. Lignin Precursor: Carbon Structure

Lignin’s intrinsic heterogeneity establishes the first level of this relationship. Kraft, organosolv, soda, and lignosulfonate lignins differ in molecular weight, degree of condensation, S/G ratio, oxygen-containing functionalities, sulfur content, ash content, and residual carbohydrate content. These characteristics influence the thermal decomposition, carbon yield, aromatization, and reactivity of the resulting char toward activation. Therefore, the same carbonization and activation conditions can produce substantially different pore structures and surface chemistries when applied to lignins from different sources [4,186]. This precursor dependence is particularly important for technical lignins because inorganic species and sulfur-containing structures may participate in carbonization and activation, acting as intrinsic dopants or catalysts for structural rearrangement.

Lignin fractionation provides an additional means of controlling this relationship between lignin and cellulose. Reducing the molecular weight distribution and selectively enriching functional groups can modify lignin reactivity before carbonization. Thus, fractionation should not be regarded simply as a purification step but as a strategy for producing a more controlled molecular precursor for carbon formation. This is consistent with a recent study showing that low-molecular-weight lignin can yield nitrogen-containing activated carbons with high surface areas and significant capacitance under appropriate activation conditions [187].

### 7.2. Carbonization/Activation and Pore Architecture

Carbonization establishes the carbon framework, whereas activation determines the extent and architecture of porosity. KOH activation generally promotes extensive micropore development and high BET surface area, while H_3_PO_4_ and other activation strategies can generate broader pore-size distributions with greater mesoporosity. Therefore, the final pore structure depends on precursor composition, carbonization temperature, activating agent type, activating agent-to-carbon ratio, and activation temperature [4,188].

However, the relationship between activation severity and electrochemical performance is not linear. Increasing activation can initially enhance charge-storage capacity by increasing accessible surface area, but excessive activation may widen or collapse pores, reduce carbon yield, remove electrochemically active surface groups, and weaken the carbon framework’s mechanical or electrical integrity. Thus, optimal activation requires balancing pore development with preservation of a conductive carbon skeleton rather than maximizing chemical etching [115,188].

The contribution of porosity to capacitance is also more complex than a direct correlation between BET surface area and charge storage. BET analysis, typically based on nitrogen adsorption at 77 K, measures gas-accessible surface area, which may differ substantially from the surface accessible to solvated electrolyte ions under electrochemical conditions. Consequently, part of the measured surface area can be electrochemically inactive or kinetically inaccessible [188].

Micropores provide a large interfacial area for electrical double-layer formation, whereas mesopores facilitate ion transport toward internal microporous regions. Macropores can act as electrolyte reservoirs and reduce diffusion distances. Therefore, a hierarchical structure combining these pore regimes can provide a better balance between capacitance and rate capability than a carbon dominated exclusively by ultramicropores [189].

This effect has been demonstrated for lignin-derived hierarchical porous carbons. Fan et al. [190] prepared carbons with similar specific surface areas of approximately 2200 m^2^ g^−1^ by varying the mesopore-to-micropore ratio. Despite comparable BET areas, their electrochemical performance differed markedly, confirming that the mesopore fraction is critical for balancing capacitance and rate performance. Therefore, BET surface area alone is an incomplete descriptor of electrochemical accessibility; pore-size distribution, connectivity, tortuosity, and compatibility between pore dimensions and electrolyte-ion size are more informative for establishing structure–performance relationships in lignin-derived activated carbons.

### 7.3. Surface Chemistry and Pseudocapacitive Charge Storage

The carbon framework provides the principal surface for EDLC charge storage, but lignin-derived carbons can also retain oxygen-containing functionalities originating from the precursor material. Phenolic, hydroxyl, carbonyl, carboxyl, and quinone-type groups can influence surface polarity and electrolyte wettability and, depending on the electrolyte and potential range, contribute to reversible faradaic reactions [191].

This creates an intrinsic processing trade-off. Higher carbonization temperatures promote aromatic condensation, structural ordering, and electrical conductivity but generally reduce oxygen-containing groups. Conversely, lower thermal severity can preserve a more chemically active surface but may result in lower conductivity and less developed carbon ordering. Therefore, the optimum electrode requires a controlled balance between electronic transport and surface redox activity, rather than simply maximizing either the carbonization severity or functional group concentration.

The incorporation of heteroatoms further modifies this relationship. Nitrogen, sulfur, phosphorus, and boron can alter electron density, defect density, wettability, conductivity, and pseudocapacitive behavior. However, the electrochemical effect depends on the dopant’s chemical configuration rather than solely on its concentration [191,192]. Recent work on nitrogen-enhanced activation is particularly relevant because it demonstrates that nitrogen functionality can influence not only the surface chemistry but also the activation process itself and the development of porosity. Yi et al. reported that nitrogen-functionalized lignin followed by NaOH activation produced a carbon with 2749 m^2^/g and 292 F/g, while statistical analysis showed that nitrogen functionality loss and activator dosage were important variables governing pore development [192].

Thus, heteroatom doping should not be considered merely an additional post-treatment designed to increase the pseudocapacitance. It can modulate the evolution of the carbon structure during activation, thereby creating a direct connection among chemical composition, pore formation, surface chemistry, and electrochemical response.

### 7.4. Structure–Performance Trade-Offs

These relationships reveal several fundamental trade-offs to consider when designing lignin-derived activated carbon electrodes. Increasing activation severity can increase the surface area but reduce the carbon yield and conductivity. Maximizing microporosity can increase the low-rate capacitance but restrict ion transport. Increasing mesoporosity can improve the rate capability but decrease the fraction of the surface available for charge accumulation. Retaining oxygen or heteroatom functionalities can enhance wettability and pseudocapacitance while potentially decreasing electrical conductivity and thermal stability [191].

Accordingly, the highest-performing material is not necessarily the one with the highest BET surface area, heteroatom content, or degree of graphitization. Instead, optimal performance arises from a balanced architecture in which the porosity, surface chemistry, and electronic conductivity are simultaneously optimized. This is increasingly supported by recent studies on lignin-derived carbons, in which materials with comparable surface areas exhibit substantially different capacitance and rate capability due to differences in their hierarchical pore structures [190].

## 8. Current Challenges and Future Perspectives

Despite the considerable progress achieved in lignin-derived activated carbons for electrochemical energy storage, their reproducibility, scalability, and practical implementation remain challenging. A fundamental limitation is the structural heterogeneity and polydispersity of lignin, whose H, G, and S units, interunit linkages, molecular weight distribution, degree of condensation, and functional group composition vary substantially with biomass origin and extraction process. The differences among kraft, organosolv, soda, and sulfite lignins consequently affect thermal decomposition, carbon yield, graphitization, and activation behavior. At the same time, inorganic constituents and process-derived species, such as ash, sulfur, and sodium, can further modify the carbonization pathways. This variability complicates comparisons between studies and contributes to batch-to-batch differences in electrode properties [193,194,195]. Recent studies on lignin-derived hard carbons have further demonstrated that precursor molecular characteristics influence the formation of open and closed pores and, consequently, sodium storage behavior, highlighting the importance of controlling lignin before carbonization [195,196].

The second challenge is achieving an appropriate balance between the carbon structural ordering, electrical conductivity, carbon yield, and surface functionality. Thermal conversion requires dehydration, cleavage, condensation, aromatization, and deoxygenation of the native lignin structures. Increasing the carbonization temperature generally promotes more ordered carbon domains and higher conductivity; however, excessive thermal treatment can reduce the carbon yield and remove oxygen-containing groups that contribute to wettability and faradaic charge storage. Therefore, future strategies should focus on controlling the carbonization pathway through precursor fractionation, optimized heating profiles, templating, and catalytic graphitization, rather than relying solely on higher temperatures [191,193]. Similarly, heteroatom incorporation (N, P, S, and B) can modify the electronic structure, wettability, defects, and redox activity, but its effectiveness depends on the dopant’s chemical configuration; therefore, controlled functionality should be prioritized over maximizing total heteroatom content [189,191].

Pore architecture is another critical challenge. Although a high BET surface area has traditionally been associated with high electrochemical performance, charge storage depends more directly on pore accessibility, connectivity, and compatibility with electrolyte ions. Excessive ultramicroporosity restricts ion transport, whereas insufficient mesoporosity limits the rate capability. Conversely, excessive mesoporosity may reduce the surface area available for double-layer formation. Thus, the optimal pore architecture depends on the electrolyte, ion size, solvation structure, and operating conditions [195,196]. Chemical activation with KOH, NaOH, H_3_PO_4_, or ZnCl_2_ remains effective for generating hierarchical porosity; however, its chemical consumption, washing requirements, water demand, and waste generation can hinder large-scale implementation. Therefore, future approaches should focus on controlled pore engineering with lower chemical and energy requirements through physical or catalytic activation, templating, self-activation, and integrated processing routes [4,194,197].

Another barrier is the transition from high-performance laboratory materials to practical devices. The specific capacitance measured in three-electrode configurations does not necessarily translate into device-level performance, which depends on active material loading, electrode thickness, electrolyte, voltage window, electrode balancing, and cell configuration. Future studies should therefore emphasize two-electrode devices and realistic mass loadings while systematically reporting the energy and power density, equivalent series resistance, rate capability, and long-term cycling stability. Similar considerations apply to lignin-derived hard carbons for sodium-ion batteries, where the initial Coulombic efficiency, reversible capacity, electrode density, and compatibility with practical electrolytes remain critical parameters [195,198]. In this context, precursor engineering and lignin fractionation are promising strategies. Membrane separation, organosolv processing, ionic liquids, and deep eutectic solvents can generate fractions with more controlled molecular weight distributions, functional group compositions, and inorganic contents, enabling more predictable carbonization and activation behavior. Recent evidence that lignin molecular fractionation can regulate the pore structure and electrochemical properties of hard carbon reinforces the importance of precursor engineering as a complement to post-carbonization activation [195,196].

The application scope of lignin-derived carbon can be expanded using hybrid electrode architectures and next-generation batteries. Lignin-derived carbons can serve as conductive, porous frameworks for metal oxides, hydroxides, conducting polymers, graphene-related materials, and other redox-active phases, thereby combining electrical double-layer and Faradaic storage mechanisms [189,199]. In sodium-ion batteries, lignin-derived hard carbon is particularly attractive because its disordered structure, interlayer spacing, defects, and open/closed pore populations can be tailored through precursor selection and thermal treatments. Recent studies have shown that controlling lignin molecular fractions can regulate closed-pore formation and, consequently, influence the plateau capacity for sodium storage [196]. However, further progress will require improvements in the initial Coulombic efficiency, electrode density, rate capability, structural stability, and full-cell performance.

Finally, the development of lignin-derived activated carbon must be evaluated from both environmental and economic perspectives. The renewable origin of lignin does not automatically ensure a lower environmental footprint, since carbonization temperature, activation chemistry, solvent and water consumption, washing, energy demand, carbon yield, chemical recovery, and transportation all contribute to the overall environmental impact. Therefore, life-cycle assessment and techno-economic analysis should be integrated with electrochemical evaluation, preferably within forest or agricultural biorefinery systems, in which lignin is valorized alongside other biomass-derived products [194,199]. Overall, the future of lignin-derived activated carbon should move beyond the pursuit of record BET surface areas or specific capacitances toward the integrated control of precursor chemistry, carbonization, pore architecture, surface functionality, electrolyte compatibility, device performance, scalability, and environmental impact. The key scientific challenge is to establish predictive and transferable relationships between lignin molecular structure, processing history, carbon architecture, and electrochemical behavior, thereby enabling the rational design of materials for specific electrolytes, storage mechanisms, and device configurations, rather than the continued empirical search for isolated high-performance materials [193,195,200].

## 9. Conclusions

Lignin, long regarded as a low-value by-product of the pulp and paper industry, has emerged over the past decade as a versatile and abundant renewable feedstock for functional carbon materials with genuine potential in electrochemical energy storage applications. As reviewed here, its aromatic, highly cross-linked structure and the diversity introduced by different biomass sources and isolation methods (Kraft, organosolv, soda, and lignosulfonate) directly shape the physicochemical properties and ultimately the performance of the carbons obtained through pyrolysis, hydrothermal carbonization, physical or chemical activation, and heteroatom doping. Judicious control of these thermochemical routes converts lignin into porous, conductive carbon architectures that rival, and in several reported cases exceed, the specific capacitance and cycling stability of carbons derived from petroleum-based or other biomass precursors, while also serving as active or auxiliary components in batteries, hybrid supercapacitors, and other electrochemical energy storage devices.

Beyond their electrochemical performance, lignin-derived carbon materials offer the chance to go beyond sustainable energy storage by converting an underutilized industrial residue into high-value functional material. Inclusion of lignin in electrochemical devices may reduce dependence on fossil carbon precursors, allow renewable carbon to be used more efficiently, and support circular economy strategies within integrated biorefinery systems. But environmental advantages of lignin valorization cannot be assumed solely from its renewable origin: they depend strongly on extraction routes, chemical consumption, energy demand, solvent recovery, and overall life cycle impact of the conversion process. Thus, future developments should combine electrochemistry optimization with sustainability assessments, so lignin-based technologies deliver real environmental benefits.

Simultaneously, this review shows that the field has not yet resolved a set of interlocking scientific and practical problems: feedstock and process heterogeneity limit reproducibility, activation chemistry carries a significant environmental burden, techno-economic margins remain narrow, and the structural disorder long treated as a liability may be central to superior capacitive performance. None of these challenges appear insurmountable; however, overcoming them will require an integrated approach that combines fundamental structure–property research, greener and more scalable processing, data-driven material design, and stronger links to biorefineries that already produce lignin at scale. Framed in these terms, lignin-derived carbon materials are not merely a niche academic curiosity but a credible and scalable pathway toward more sustainable electrochemical energy storage, which converts an existing industrial residue into a strategic resource for the ongoing transition to a circular, low-carbon energy economy.

The sustainable value of lignin-derived carbon materials will therefore depend not only on achieving high electrochemical performance but also on establishing environmentally responsible processing pathways and demonstrating advantages over conventional materials through holistic techno-economic and life-cycle evaluations. Framed in these terms, lignin-derived carbon materials are not merely a niche academic curiosity but a credible pathway toward more sustainable electrochemical energy storage, converting an existing industrial residue into a strategic renewable resource for the transition toward a circular, low-carbon energy economy.

## Figures and Tables

**Table 1:** Comparison of technical lignins: production process, structural characteristics, physicochemical properties, and relevance for electrochemical applications.

Characteristic	Kraft Lignin	Organosolv Lignin	Lignosulfonate	Soda Lignin
Production process	Alkaline cooking with NaOH/Na_2_S at high temperature and pH; lignin recovered from black liquor (e.g., LignoBoost, LignoForece) [41]	Delignification in organic solvents (commonly aqueous ethanol, but also acetic acid, etc.) with or without acid catalyst; often at milder conditions than kraft [41,42,43]	Sulfite pulping (bisulfite/SO_2_ in neutral–acidic conditions); lignin sulfonated and dissolved as lignosulfonate salts [44,45]	Sulfur-free alkaline pulping with NaOH (sometimes with anthraquinone) under kraft-like conditions [46].
Sulfur Content	Typically 1–3wt% (as thiol/sulfide-related structures) [45]	Essentially sulfur-free (unless mixed processes); advantage for high-value applications [41,46].	High, due to covalent –SO_3_^−^ groups (sulfonic acids) introduced during pulping; S contents ~4–7 wt% are typical [45].	Essentially zero; key advantage for some applications (e.g., nutrition, certain resins) [45].
Molecular weight	Mw usually ~1000–5000 g/mol, broad and heterogeneous; strong condensation under harsh conditions increases Mw and polydispersity [44,45].	Generally lower Mw and lower polydispersity than kraft; meta-analyses show Mw decreases with increasing extraction severity (temperature, acid concentration, time) [40,43].	High Mw, often 20,000–50,000 g/mol, with high polydispersity [45].	Typically in the same broad range as kraft lignin (~800–3000+ g/mol), but values depend strongly on feedstock (non-wood vs. wood) and process severity [40,45].
Solubility	Low water solubility; soluble under alkaline conditions	Soluble in organic solvents and alkaline solutions; hydrophobic in water.	Highly water-soluble over a wide pH range due to sulfonate groups.	Similar to kraft—insoluble in neutral water, soluble in alkaline media.
Ash and inorganic impurities	Moderate ash and carbohydrate impurities; depends strongly on isolation process (typically 1–3% ash) [44,47]	Low ash, low carbohydrate contamination; often close to “native-like” lignin, attractive for materials and fine chemicals [41,42,43].	Often high ash and inorganic content from counter-ions (Na^+^, Ca^2+^, etc.) [44,45]	High (sulfur-free)
β–O–4 structure	Heavily cleaved; kraft lignin is enriched in condensed structures and phenolic OH, and depleted in aliphatic OH compared to native lignin [44,48]	Typically exhibits the highest retention of β–O–4 linkages among common technical lignins [43,48].	Partially preserved; less cleavage than kraft, but sulfonation and some condensation occur [44]	Presence of vinyl ether structures and other alkaline-derived units; moderate preservation of β–O–4 linkages—less degraded than kraft but more than Organosolv [46].
Electrochemical relevance	Carbon precursor; high carbon yield	High β–O–4 preservation; tunable carbon materials	Water dispersibility; binders/electrolyte interfaces	Sulfur-free carbon precursor

**Table 2:** Comparison of chemical modification strategies for lignin and their effects on material properties.

Chemical Modification Strategy	Major Transformation	Resulting Property Changes	Reactive Sites
Carboxymethylation	–CH_2_COO^−^ introduction	↑ negative charge, hydrophilicity, coordination	Phenolic/aliphatic OH groups
Sulfomethylation	–CH_2_SO_3_^−^ introduction	↑ negative charge, solubility, dispersibility	Reactive aromatic positions
Esterification	OH → ester conversion	↑ hydrophobicity, organic solubility, flexibility and processability	Phenolic and aliphatic OH groups

The arrows in the table indicate the following: upward arrows (↑) represent an increase, while rightward arrows (→) denote conversion or an effect.

**Table 3:** Carbonization, activation, doping, and graphitization strategies for lignin-derived carbon materials.

Carbon Modification Strategy	Activation Agent/ Processing Conditions	Structural Modification	Resulting Material Property	References
Physical activation	CO_2_/steam, 700–900 °C	Selective gasification of edges and defects	Moderate microporosity	[111,128]
Chemical Activation (KOH)	KOH, 600–900 °C	Potassium intercalation and redox-assisted carbon gasification	High surface area (Up to 2842 m^2^ g^−1^)	[114]
Chemical Activation (H_3_PO_4_)	H_3_PO_4_, 400–500 °C	Phosphate-mediated crosslinking and temporary templating	Enhanced mesoporosity	[116]
Comparative activation study	KOH/ZnCl_2_/CO_2_	Activation-dependent evolution of carbon microstructure	Direct comparison of pore development	[120]
Heteroatom doping (N,S)	Self-assembly + coupled activation	N/S redox sites in a hierarchical matrix	Improved capacitance performance	[124]
Nitrogen doping	Co-pyrolysis with N precursor	Pyridine/pyrrole species (XPS)	Microporosity + Stable N	[122]
Dual heteroatom doping (N,O)	Dual heteroatomic functionalization	Greater affinity for electrolyte cations	Supercapacitor + CO_2_ capture	[129]
Catalytic graphitization	Fe, Ni, Co, Cu; 900–1100 °C	Dissolution-precipitation of C; graphene domains	Improved graphitization; Fe and Ni highly effective catalysts	[127]

**Table 4:** Relationship between lignin precursor, carbon conversion strategy, structural characteristics, and electrochemical performance of lignin-derived carbon materials.

Lignin Precursor/ Carbon Source	Carbonization/ Activation Strategy	Resulting Carbon Structure	Electrochemical Evaluation	Electrochemical Performance	Key Insight and Limitation
Enzymatically hydrolyzed lignin	Single-step KOH Activation (optimized LAC800-4)	Hierarchical interconnected porous carbon with high specific surface area (2285 m^2^ g^−1^)	Three-electrode system and symmetric supercapacitor cell	291.3 F/g (three- electrode); 186.8 F/g (symmetric cell); 96.1% capacitance retention after 10,000 cycles; 6.5 Wh/kg	High electrode capacitance does not necessarily translate into equivalent device performance. Ion accessibility and cell configuration strongly influence practical capacitance.
Lignin micelles + sulfomethylated melamine	MgCO_3_ templating combined with N,S co- doping	Hierarchical porous carbon with high surface area (2538.3 m^2^ g^−1^) and N,S-containing surface functionalities	Supercapacitor electrode evaluation	393 F/g at 0.5 A/g	Enhanced performance results from the combined contribution of hierarchical porosity and heteroatom functionalities; however, individual effects of templating and doping cannot be fully separated.
Lignosulfonate + Fe^3+^-assisted carbon precursor	Self-assembly without chemical activation or external template	N,S-doped carbon (8.47% N; 2.56% S) generated through precursor- assisted self- assembly	Supercapacitor evaluation	390 F/g; 14.7 Wh/kg	High electrochemical performance can be achieved without traditional activation, highlighting the role of precursor chemistry, heteroatom configuration, and accessible surface structure.
Lignin-derived carbon	KOH activation with increasing activation severity	Progressive micropore development, surface area increase, and possible pore widening at excessive severity	Electrochemical energy storage evaluation	Surface areas up to 2842 m^2^/g reported	Increasing activation improves porosity but excessive KOH promotes carbon loss and over-gasification, creating a trade-off between surface area and structural preservation.
Lignin-derived carbon	H_3_PO_4_ activation (~400–600 °C)	Broader pore- size distribution with increased mesoporosity; surface areas >2000 m^2^/g reported	Electrochemical energy storage evaluation	Performance depends on resulting pore architecture	Compared with KOH activation, H_3_PO_4_ favors more open pore structures, which may improve ion transport depending on electrolyte and device requirements.
Lignin-derived carbon	Physical activation (CO_2_/steam) versus chemical activation (KOH, ZnCl_2_, H_3_PO_4_)	Different micro/mesopore ratios and surface functional group distributions	Supercapacitor-type charge storage evaluation	Not directly comparable across studies	Activation strategy simultaneously modifies porosity, surface chemistry, and carbon yield; therefore, performance comparisons require consideration of multiple structural parameters.
KOH-activated lignin carbon	Moderate Activation (~800 °C)	Balanced micropore development with preservation of mesoporous pathways	High-rate supercapacitor evaluation	Improved ion transport and rate capability reported	Maximizing surface area is not always optimal; balanced pore accessibility and ion diffusion are critical for high-current applications.

**Table 5:** Electrochemical performance of lignin-derived carbon materials compared with conventional lithium-ion battery components.

Material/System	Lignin-Derived Design Strategy	Role in Battery System	Electrochemical Performance	Testing Conditions/ Relevant Characteristics	References
Rice-straw lignin-derived carbon nanofibers	Carbon nanofiber formation from lignin precursor for conductive porous carbon architecture	Anode active material	Initial discharge capacity: 1108.3 mAh g^−1^; first-cycle reversible capacity: 377.3 mAh g^−1^; capacity retention: 84.6%	84.6% capacity retention after 100 cycles at 1C	[164]
Chloroacetic-acid- modified lignin porous carbon	Chemical functionalization of lignin followed by porous carbon formation	Anode active material	Specific capacity: 500 mAh g^−1^; 99% capacity retention	Tested at 50 mA g^−1^; cycling stability evaluated at 1000 mA g^−1^ for 1000 cycles	[165]
Natural lignin- derived porous carbon	Direct conversion of lignin into porous carbon structure	Anode active material	Specific capacity: 420 mAh g^−1^; 99% capacity retention	Tested at 0.2 A g^−1^; 300-cycle evaluation	[167].
Commercial graphite	Conventional graphitic carbon anode benchmark	Reference anode material	Theoretical capacity: 372 mAh g^−1^; reversible capacity ≈350 mAh g^−1^	LiC_6_ represents the fully lithiated graphite phase; graphite/PVDF electrode configuration	[169,170]
PVDF binder	Conventional polymeric binder system (not lignin-derived)	Electrode binder	Not an active capacity-bearing component	Widely used binder in graphite-based lithium-ion battery electrodes	[171]
Commercial PE/PP separator	Conventional microporous polymer membrane (not lignin-derived)	Separator component	Not an active capacity-bearing component	PE, PP, or PP/PE/PP multilayer membranes used for ion transport and electrode separation	[172]

## Data Availability

No new data were created or analyzed in this study. Data sharing is not applicable to this article.

## Abbreviations

The following abbreviations are used in this manuscript:

EES	Electrochemical energy storage
H	p-hydroxyphenyl unit
G	Guaiacyl unit
S	Syringyl unit
NMR	Nuclear magnetic resonance
HTC	Hydrothermal carbonization
FTIR	Fourier Transform Spectroscopy
TGA	Thermogravimetric analysis
DSC	Differential Scanning Calorimeter
GC/MS	Gas chromatography–mass spectrometry
Py-GC/MS	Pyrolysis–gas chromatography–mass spectrometry
Mw	Molecular weight
PDI	Polydispersity index
BET	Brunauer–Emmett–Teller
